# Zinc-encapsulating covalent organic frameworks for enhanced chemiresistive NH_3_ sensing at room temperature†

**DOI:** 10.1039/d5ra01430a

**Published:** 2025-05-19

**Authors:** Sujith Benarzee Nallamalla, Naresh Kumar Katari, A. Jagan Mohan Reddy, Sreekantha Babu Jonnalagadda, Surendra Babu Manabolu Surya

**Affiliations:** a Department of Chemistry, GITAM University Hyderabad-502329 Telangana India smanabol@gitam.edu; b School of Chemistry & Physics, College of Agriculture, Engineering & Science, Westville Campus, University of KwaZulu-Natal P Bag X 54001 Durban 4000 South Africa; c Departments of Chemistry, CMR Technical Campus Medchal Hyderabad 501401 India

## Abstract

Ammonia (NH_3_) is a hazardous gas used in industry, agriculture, and biomedical applications, and the development of efficient room-temperature and low-concentration ammonia detection sensors is essential. However, conventional sensors, including metal oxides, nanocomposites, and MOFs, require highly elevated temperatures (200–500 °C), leading to high energy consumption and less durability. To overcome these challenges, we developed functionalized zinc-encapsulated covalent organic frameworks (Zn@COFs) using a facile metal-doping approach. COFs doped with zinc have a modulated electronic environment, increased active sites, efficient charge transfer, and enhanced gas interactions. The incorporation of Zn^2+^ into the COF frameworks was confirmed by IR, TEM-EDAX, ^13^C CP MAS NMR spectra (C=O peak at ∼183 ppm, and imine C=N peaks at ∼148 and ∼146 ppm) and XPS (C=O peak at 527.84 eV, C=N at 399.2 eV; Zn 2p_3/2_ peak at 1042 eV, and Zn 2p_1/2_ at 1019 eV). Among the synthesized frameworks, Zn@COF-3 exhibited exceptional NH_3_ sensing at a concentration of 1 ppm at room temperature, with a rapid response time (26 s) and recovery time (18 s), outperforming pristine COFs and Zn@COFs. This superior performance is attributed to its rich active sites (C=O), high surface area (335 m^2^ g^−1^), porosity, strong NH_3_ adsorption energy (−282 kJ mol^−1^), and low energy gap (2.65 eV), as confirmed by DFT calculations. Additionally, Zn@COF-3 shows excellent selectivity and long-term stability over 30 days. This Zn@COF-based approach yields next-generation ammonia sensors, featuring energy-efficient, highly selective, and room-temperature chemiresistive sensors for industrial, environmental, and biomedical applications.

## Abstract 2

A Zn-encapsulating COF sensor (Zn@COF-3) fabricated *via* a drop-casting method demonstrates a selective and rapid chemiresistive response to NH_3_ gas at room temperature, making it suitable for real-time gas sensor applications.

## Graphical abstract

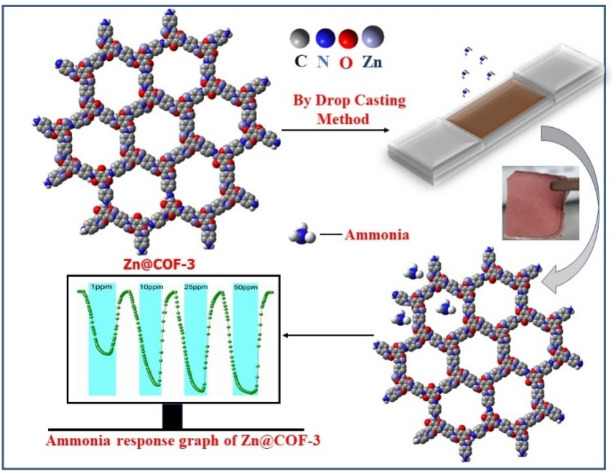

## 1. Introduction

Gas sensors are gaining significance in contemporary society for detecting harmful gasses in various conditions, including the environment, industrial facilities, residential areas, and public spaces.^1–3^ These sensors are essential in various applications, such as toxic gas monitoring in public places, air quality control, safety systems, and biomedical diagnostics.^4,5^ Chemiresistive gas sensors are becoming increasingly popular due to their simple design, capacity to monitor continuously, flexibility, and ease of connection with ordinary electrical systems.^6,7^

Ammonia (NH_3_) is a significant industrial chemical with widespread applications, but it poses severe health and environmental risks. Exposure to NH_3_ at concentrations exceeding 300 ppm can result in significant irritation to the eyes and skin, along with burning sensations in the nasal passages, throat, and respiratory tract, potentially causing cellular damage within the body. Ammonia leaks in industrial production indeed pose significant hazards, with concentrations between 15% and 28% by volume being capable of creating flammable and potentially explosive environments. The Occupational Safety and Health Administration (OSHA) has established allowable exposure limits of 25 ppm for an 8 h duration and 35 ppm for a 15 min interval, which is above the olfactory detection threshold of roughly 20 ppm.^8,9^ Moreover, ammonia functions as a crucial biomarker in breath diagnostics for identifying renal disorders, with increased concentrations (exceeding 800 ppb) signifying health complications. Despite thorough investigation, metal-oxide-based sensors, which are frequently employed for NH_3_ detection, have been constrained by their limited detection range and generally necessitate elevated operating temperatures (100 °C to 300 °C).^10–12^ Two-dimensional materials like carbon nanotubes (CNTs) and metal oxides such as TiO_2_, ZnO, and WO_3_ have gained considerable attention in gas sensor technology due to their adaptable structural, physical, and chemical properties.^13–17^ These materials, which are categorized as either n-type or p-type semiconductors, play a crucial role in gas sensing applications by facilitating charger transfer interactions upon exposure to target gases. Notably, they exhibit optimal gas sensing performance within the temperature range of 250–550 °C.^18–20^ Hence, it is essential to study new sensing materials that can effectively detect ammonia at lower temperatures and with enhanced sensitivity and selectivity. Therefore, the development of excellent sensors for NH_3_ detection at ambient temperature remains a significant challenge.

COFs are a promising new class of materials for next-generation gas sensors and have attracted a great deal of attention since the groundbreaking investigation due to their unique characteristics, which include adjustable porosity, an organic backbone, and remarkable stability.^21^ Compared to metal–organic frameworks (MOFs), COFs exhibit superior chemical and thermal stability, making them more suitable for long-term sensing under humid conditions.^22–24^ MOFs often suffer from framework collapse due to weak metal–ligand interactions in humid or high-temperature environments due to the metals present in the framework being converted into metal oxides at high temperature, whereas COFs, which are built on strong covalent linkages, provide greater long-term stability and reusability in gas sensing applications. In comparison to MOFs, COFs have shown potential in diverse chemical sensing applications^25–30^ such as explosive detection,^31,32^ detection of metal ions,^33,34^ humidity detection,^35,36^ pH levels,^37,38^ and gases.^39,40^ However, pristine COF-based sensors still face some limitations, such as insolubility, weak conductivity, less active sites, more aggregation, weak electronic environment, and poor dispersion of microcrystalline nature.^41–49^ To overcome these challenges, adding metal or metal oxide NPs into COFs is a key way to create a strong synergistic interaction between the framework and the metal, which results in better gas-sensing results.^50–54^ Metal covalent organic frameworks (MCOFs), which are formed by incorporating metal ions into COF structures, present a promising advancement in material design, as the metal centers act as catalytic and adsorption sites, significantly improving sensitivity and selectivity for various gases.^55^

While MCOFs provide enhanced gas-sensing properties, the choice of the dopant metal significantly influences their performance. Among the various metal dopants, Zn^2+^ has been selected for COF modification due to its ability to enhance charge transfer, improve conductivity, and maintain structural integrity.^56^ Unlike other transition metals, the incorporation of Zn^2+^ preserves the porosity of the COF, ensuring efficient gas diffusion while introducing Lewis acid sites that strengthen interactions with NH_3_.^57^ Its superior chemical stability, non-toxic nature, and proven effectiveness in gas sensing make it an ideal choice for enhancing COF-based NH_3_ and VOC detection.^58^ Zn^2+^ has been selected over precious metals such as Pt, Au, or Pd due to its low cost and non-toxic nature. Additionally, it facilitates strong charge transfer, offers a high surface area, introduces Lewis acid sites for NH_3_ adsorption, and enables effective operation at ambient conditions. Furthermore, Zn^2+^ exhibits fast response and recovery times along with long-term chemical stability, making it highly suitable for gas-sensing applications.^59^ COFs' uniform and stable porous structure also makes them ideal for single-atom catalysts (SACs), as they can be tailored through a bottom-up synthesis approach and post-metalation to introduce specific catalytic sites.^60–62^

In this work, we focus on the synthesis of a series of covalent organic frameworks *via* the controlled increase of hydroxyl (–OH) groups in Ar(CHO)_3_, which directs the formation of β-keto-enamine COFs (COF-1, COF-2, and COF-3). This step-wise functionalization results in COF-1 with a single β-keto-enamine group, COF-2 with two β-keto-enamine groups, and COF-3 with three β-keto-enamine groups. Following the successful synthesis of the COFs, they are subsequently doped with Zn^2+^ ions to enhance their gas-sensing performance (Zn@COF-3 at 1 ppm, *T*_res_ = 26 s and *T*_rec_ = 16 s in NH_3_ gas sensing). Additionally, density functional theory (DFT) calculations using the B3LYP method were performed to analyze the electrostatic potential (ESP), highest occupied molecular orbital (HOMO), and lowest unoccupied molecular orbital (LUMO) of the COFs and MCOFs, providing insights into their interactions with NH_3_ molecules. The findings from this study highlight the effectiveness of Zn^2+^ doping and progressive functionalization in enhancing the sensing performance of COFs, demonstrating their potential for real-world gas detection applications.

## 2. Experimental section

### 2.1 Synthesis of parent COFs

**Fig. 1:**
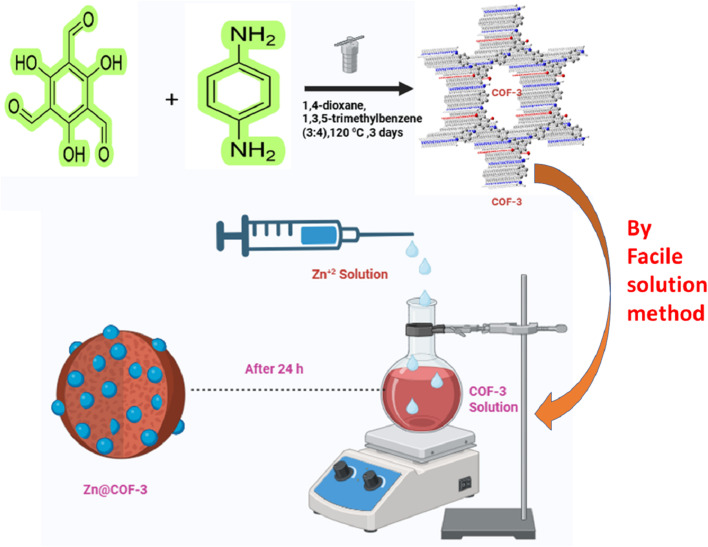
Synthetic procedure for covalent organic frameworks (COF-1, 2, and 3) and metal-encapsulating COFs (Zn@COF-1, Zn@COF-2, and Zn@COF-3).

The synthesis of COF-1, COF-2, and COF-3 was carried out by Schiff-base condensation of 2-hydroxy-1,3,5-benzenetricarbaldehyde, 2,4-hydroxy-1,3,5-benzenetricarbaldehyde, or 2,4,6-hydroxybenzene-1,3,5-tricarbaldehyde with 1,4-benzenediamine to form the respective COFs (Fig. 1).

#### 2.1.1 Synthesis of COF-1

COF-1 was synthesized using the procedures stated in the literature, with a few modifications.^62–64^ In a round bottom flask, 2-hydroxy-1,3,5-benzenetricarbaldehyde (73 mg), 1,4 benzenediamine (58 mg), 1.5 mL of 1,4-dioxane, 2.0 mL of 1,3,5-trimethylbenzene, and 0.3 mL of 6 M aqueous acetic acid solution were mixed uniformly. The solution was subjected to sonication for 30 minutes under a nitrogen (N_2_) atmosphere. Subsequently, the mixture was moved to an autoclave, placed in a hot air oven at 130 °C, and maintained for 3 days. After cooling, the final product was washed with acetone, hexane, and anhydrous THF.^65^ Subsequently, vacuum-drying at a temperature of 100 °C for 24 h was carried out, resulting in the formation of COF-1.

#### 2.1.2 Synthesis of COF-2

Similarly, COF-2 was synthesized using a similar procedure, with slight modifications to the aldehyde precursor. Initially, the round bottom flask contained 2,4,-hydroxy-1,3,5-benzenetricarbaldehyde (73 mg), 1,4-benzenediamine (58 mg), 1.5 mL of 1,4-dioxane, 2.0 mL of 1,3,5-trimethylbenzene, and 0.3 mL of 6 M aqueous acetic acid. The solution was sonicated under a nitrogen atmosphere and transferred into a stainless-steel Teflon autoclave container maintained at 130 °C for three days and then cooled to room temperature. Purification and drying processes were carried out, similar to the COF-1 process, leading to the formation of COF-2.

#### 2.1.3 Synthesis of COF-3

In a round bottom flask, 73 mg of 2,4,6-hydroxybenzene-1,3,5-tricarbaldehyde, 58 mg of 1,4-benzenediamine, 1.5 mL of 1,4-dioxane, 2.0 mL of 1,3,5-trimethylbenzene, and 0.3 mL of 6 M aqueous acetic acid were mixed and sonicated in a nitrogen atmosphere. The solution was transferred into a stainless-steel Teflon autoclave container, heated at 130 °C for three days, and cooled to room temperature slowly. The final product was washed with acetone, hexane, and anhydrous THF and dried under vacuum at 100 °C for 24 h, forming COF-3.

### 2.2 Synthesis of zinc-encapsulating COFs (Zn@COFs)

The COFs (COF-1, COF-2, and COF-3) were modified with Zn^2+^ ions through a facile solution method to enhance their stability and gas-sensing capabilities. The synthesis procedures for the metal-modified frameworks Zn@COF-1, Zn@COF-2, and Zn@COF-3, are given below.

#### 2.2.1 Synthesis of Zn@COF-1

Initially, COF-1 (30 mg) was dissolved in 50 mL of distilled water to form a COF-1 suspension and transferred into the round bottom flask. In another beaker, 60 mg of ZnCl_2_ was dissolved in 50 mL of distilled water. A two-channel syringe pump was used to add ZnCl_2_ solution at a steady rate of 1 mL h^−1^ to the round bottom flask containing the COF-1 suspension. Simultaneously, the mixture was agitated using a magnetic stirrer at ambient temperature for 24 h. Afterward, the product was filtered and rinsed sequentially with hexane, acetone, and anhydrous THF to remove any unreacted precursors and by-products.^65^ The sample was further dried at a temperature of 120 °C in a vacuum for 12 h, forming Zn@COF-1.

#### 2.2.2 Synthesis of Zn@COF-2

The previously obtained COF-2 (30 mg) was dissolved in 50 mL of distilled water, Then, 50 mL of ZnCl_2_ (60 mg) aqueous solution was slowly added using a two-channel syringe pump at a steady rate of 1 mL h^−1^. Simultaneously, the mixture was agitated using a magnetic stirrer at ambient temperature for 24 h. Afterward, the product was filtered and rinsed sequentially with hexane, acetone, and anhydrous THF to remove any unreacted precursors and by-products. The sample was further dried at a temperature of 120 °C in a vacuum for 12 h, forming Zn@COF-2.

#### 2.2.3 Synthesis of Zn@COF-3

The COF-3 (30 mg) was suspended in 50 mL of distilled water. Then, 50 mL of ZnCl_2_ (60 mg) aqueous solution was slowly added using a two-channel syringe pump at a steady rate of 1 mL h^−1^. Simultaneously, the mixture was stirred for 24 h with a magnetic stirrer at ambient temperature. Afterward, the product was filtered and rinsed sequentially with hexane, acetone, and anhydrous THF to remove any unreacted precursors and by-products. The sample was further dried at a temperature of 120 °C in a vacuum for 12 h, forming Zn@COF-3. Using solvents with very low surface tension, such as hexane, makes it possible to activate the COF easily while minimizing pore collapse.^66^

### 2.3 Gas sensor fabrication and measurement

**Fig. 2:**
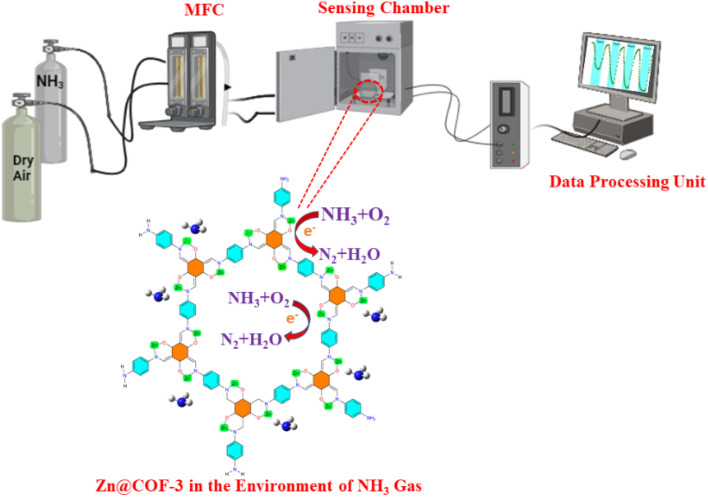
Schematic illustration of ammonia gas sensing instrument setup.

To establish the reproducibility of the pristine COF and Zn@COF-based gas sensors, multiple independent sensors were fabricated and tested under identical conditions. The fabrication of the Zn@COFs and pristine COFs was followed by their dispersion in an agate mortar, to which polyvinylidene difluoride (PVDF) was added as a binding agent. To ensure uniform dispersion, a small amount of anhydrous ethanol was added dropwise while continuously grinding the mixture for 10 min. The obtained homogeneous paste was then carefully coated onto 1 cm × 2 cm glass substrates to form thin sensing films. The coated films were dried at room temperature for 30 min to remove residual solvent and enhance film adhesion. The fabricated Zn@COFs and COF-based sensors underwent an aging process for 72 h using Winsen's TS-64B sensor gas-sensitive aging platform to enhance sensor stability and achieve a consistent response. This process is crucial for stabilizing the sensing material, ensuring minimal variations in response during subsequent measurements (Fig. 2).

Gas sensing tests were conducted at room temperature (25 ± 2 °C) under a controlled humidity of 0–75% RH with a gas flow rate of 100 mL min^−1^. Sensors were pre-exposed to dry air for 30 min before testing. NH_3_ concentrations ranged from 1 to 50 ppm, and the response was determined by the change in electrical resistance. Reproducibility was evaluated by performing multiple independent gas sensing measurements on identical Zn@COF and COF-based sensors. The response (*S*) of the sensors was calculated using the equation:

1 *S* = *R*_a_/*R*_g_

where *R*_a_ is the baseline resistance in air and *R*_g_ is the resistance upon exposure to different concentrations of ammonia gas. To validate reproducibility, four consecutive sensing cycles were conducted at an ammonia concentration of 1 ppm, and the response of each cycle was recorded. The results demonstrate minimal deviation between cycles, confirming the high reproducibility of the sensor's performance.^67^

Additionally, the long-term stability of the fabricated sensors was assessed over 30 days by exposing the sensors to 50 ppm ammonia at regular intervals. The response was monitored, and the results indicate that the sensors maintained a stable response with negligible fluctuations, highlighting the robustness and durability of the Zn@COF and COF-based materials for extended applications. As environmental humidity can influence gas sensing performance, the impact of relative humidity (RH) on the sensing response was also examined. The sensors were tested at varying humidity levels ranging from 0% to 75% RH while maintaining a constant ammonia gas concentration of 1 ppm.

### 2.4 Density functional theory calculations

With the B3LYP functional level and the 6-31G basis set, the DFT approach was utilized to optimise the geometry of Zn@COFs and perform energy calculations both with and without the NH_3_ molecule. For self-consistent field and geometry optimisation computations involving organic molecules, the 6-31G basis set is popular and efficient.^68,69^ Before computing the energy of the systems, the atoms underwent relaxation. Eqn (2) can be used to calculate the ammonia adsorption energy and binding energy on the Zn@COFs.^70–72^

2 *E*_ads(Zn@COF)_ = *E*_NH_3_/Zn@COF_ − (*E*_Zn@COFs_ + *E*_NH_3__)

Eqn (2) represents the adsorption energy of Zn@COF in the presence of NH_3_. The energies of the Zn@COF complexes are identified as *E*_NH_3_/Zn@COF_, where *E*_NH_3__ indicates the energy of a single NH_3_ molecule and *E* represents the total energy of the Zn@COF. The Gaussian 16 W program for electronic structure was used for all calculations after building the basic structure with ChemDraw.

## 3. Results and discussion

### 3.1 Structural and morphological analysis

#### 3.1.1 PXRD analysis

**Fig. 3:**
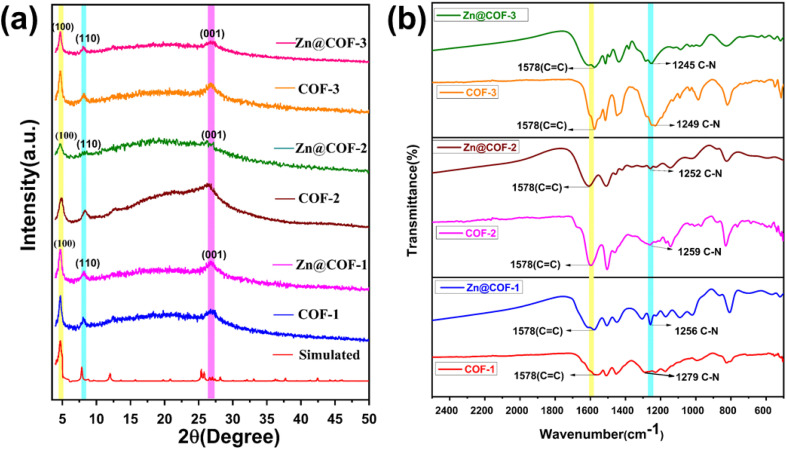
(a) Powder X-ray diffraction (PXRD) patterns of the COFs and MCOFs. (b) FTIR spectra of all COFs and MCOFs.

The PXRD patterns are shown in Fig. 3(a). The peaks at 4.8°, 8.3°, and 26.7° for the COFs (COF-1, COF-2, and COF-3) are also present in the MCOFs (Zn@COF-1, Zn@COF-2, and Zn@COF-3), and correspond to the (100), (110), and (001) planes, respectively.^62,73^ These peaks are present in both the parent and Zn-incorporating COFs, indicating that the incorporation of Zn^2+^ ions does not significantly disrupt the polycrystalline structure. The persistence of these peaks indicates that only slight alterations occur.^74^ The peak at 26.7° is caused by π–π stacking interactions between the COF molecular layers,^75,76^ and the peaks at 4.8° and 8.3° indicate a highly ordered porous structure. This means that the crystalline structure of the original COFs is maintained after the incorporation of Zn^2+^, with only minor shifts in the peak positions, likely resulting from interactions between the Zn^2+^ ions and the COF active site network.^77^ We used the *d*-spacing values from the PXRD data to determine the unit cell parameters of both the parent and Zn-modified COFs (Tables S1 and S2 ESI†). This was further supported by the X-ray diffraction (XRD) patterns of the parent COFs, which are similar to the simulated pattern.^63,78^

#### 3.1.2 FT-IR spectral analysis

Fig. 3(b) shows the FT-IR spectra of the COFs and Zn@COFs. These spectra display a significant shoulder peak at 1622 cm^−1^, which indicates the stretching of the C=O bond, and a notable peak at 1578 cm^−1.^^79^ The peak detected at 1256 cm^−1^ is caused by the stretching vibration of the C–N bond. These results indicate that the –OH group of the phloroglucinol entity is in the keto form, while the imine bond resulting from Schiff base condensation transforms into an enamine group.^80–82^ The peak corresponding to the C–N bond shifted from 1279 cm^−1^ in COF-1 to 1256 cm^−1^ in Zn@COF-1; from 1259 cm^−1^ in COF-2 to 1252 cm^−1^ in Zn@COF-2, and from 1249 cm^−1^ in COF-3 to 1245 cm^−1^ in Zn@COF-3. These changes demonstrate the coordination of Zn^2+^ to N sites.

#### 3.1.3 ^13^C CP-MAS SS NMR spectra

**Fig. 4:**
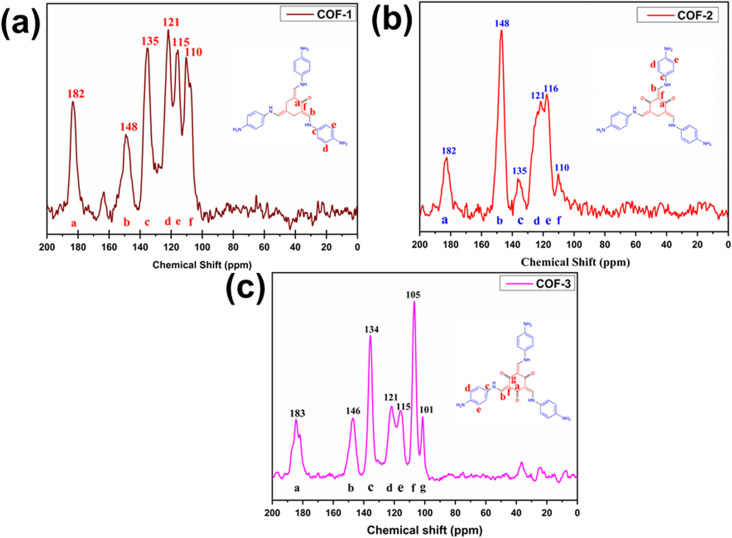
^13^C CP-MAS solid-state NMR spectra of (a) COF-1, (b) COF-2, and (c) COF-3.

The ^13^C CP-MAS SS NMR spectra confirmed the presence of imine (–C=N–) and keto (–C=O–) bonds in both the COFs and Zn@COFs, as shown in Fig. 4(a–c) and S1(a–c) (ESI†). These spectra provide insight into the chemical environment of the carbon atoms in the COF structures before and after Zn incorporation. In the pristine COFs, the characteristic peaks in the 110–150 ppm region correspond to aromatic carbon atoms, while the peaks at around 180–183 ppm (182 ppm for COF-1, 182 ppm for COF-2, and 183 ppm for COF-3) are attributed to carbonyl (–C=O–) and imine (–C=N–) groups, which play a crucial role in the framework stability and electronic properties of the COFs.^83^ Upon the incorporation of Zn, the overall peak positions remain largely unchanged, which suggests that the Zn coordination does not directly affect the carbon atoms. For Zn@COF-1 and Zn@COF-2, the peaks at 121–135 ppm exhibit slight broadening and shifting, which may indicate Zn coordination through the nitrogen atoms of the imine (–C=N–) or amine (–NH_2_) functional groups. A similar trend is observed for Zn@COF-3, for which shifts in the 105–134 ppm range further support the possibility of the interaction of Zn with nitrogen-rich sites. The absence of significant changes in the carbonyl (∼183 ppm) and aromatic (∼120–150 ppm) regions suggests that the incorporation of Zn does not strongly perturb the overall electronic structure of the COFs.^62,84^ Instead, Zn coordination likely occurs through weak interactions with nitrogen/oxygen sites, leading to localized electronic modifications rather than major structural reorganization. The lack of significant chemical shift changes after Zn incorporation is due to the non-covalent nature of Zn coordination with the COF framework. Unlike covalent modifications that strongly alter the electronic environment of carbon atoms, Zn coordination occurs through weak interactions with imine (–C=N–) and (–C=O–), leading to minimal perturbation in the carbon chemical shifts.

#### 3.1.4 FE-SEM & TEM analysis

**Fig. 5:**
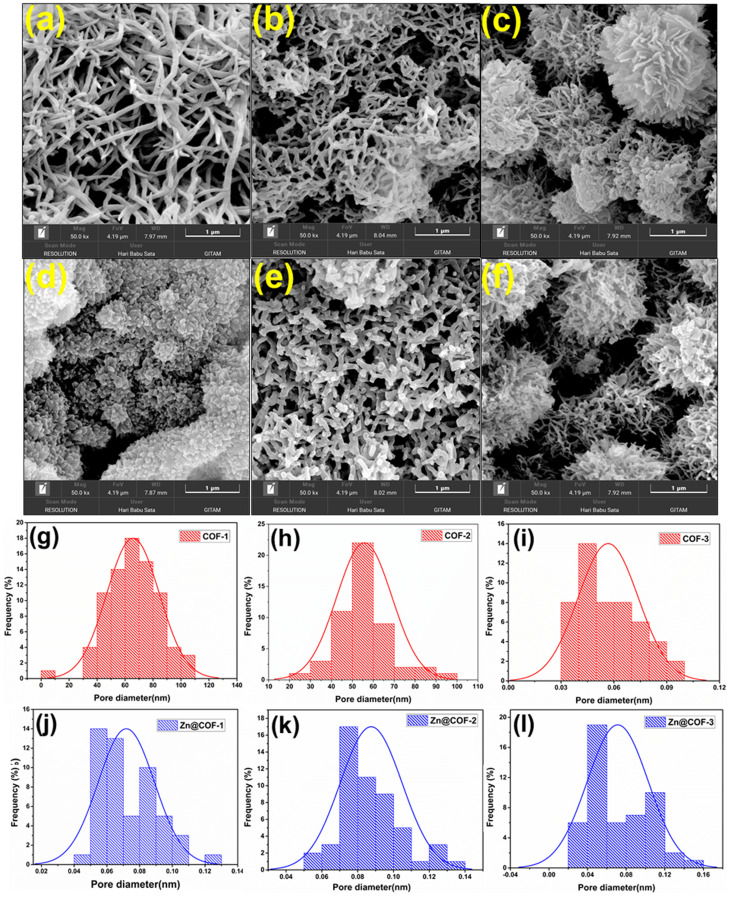
FE SEM images of (a) COF-1, (b) COF-2, (c) COF-3, (d) Zn@COF-1, (e) Zn@COF-2, (f) and Zn@COF-3. (g–i) Pore size distribution histograms of the pristine COFs ((g) COF-1, (h) COF-2, and (i) COF-3). (j–l) Pore size distribution histograms of the Zn@COFs ((j) Zn@COF-1, (k) Zn@COF-2, and (l) Zn@COF-3).

**Fig. 6:**
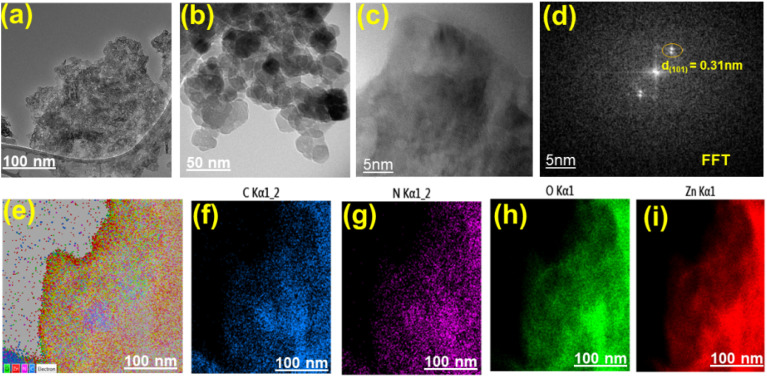
(a and b) Low resolution TEM images of Zn@COF-3. (c) High resolution TEM image of Zn@COF-3. (d) AFM 3D image of Zn@COF-3. (e–i) EDAX images of Zn@COF-3.

The SEM images of COF-1 and COF-2 (Fig. 5(a) and (b)) displayed a thread-like network morphology, which enhances the fast diffusion of gas and decreases the interaction between NH_3_ and the sensing material, while the high porosity and few active sites result in low adsorption of NH_3_. COF-3 (Fig. 5(c)) exhibited a micro-flower morphology, with increased surface area and a greater number of NH_3_ adsorption sites due to its hierarchical roughness and layered nanosheets, which helps to increase charge transfer interaction and NH_3_ interaction. The morphology of the MCOFs is similar to that of the COFs, except in the case of COF-1. Zn@COF-1 and Zn@COF-2 (Fig. 5(d) and (e)) exhibited a porous and fibrous network morphology, facilitating rapid gas diffusion, whereas Zn@COF-3 (Fig. 5(f)) showed a clustered, micro-flower-like structure with increased surface roughness, contributing to enhanced NH_3_ adsorption and charge transfer efficiency. The pore size distribution histograms (Fig. 5(g–l)) provide further insights into the porosity of the synthesized COFs and Zn@COFs. The pristine COFs (Fig. 5(g–i)) exhibit relatively uniform pore size distributions, with COF-1 and COF-2 exhibiting well-defined porosity, which promotes gas diffusion. COF-3 shows a broader pore distribution, which could be attributed to its hierarchical nanosheet arrangement. The changes in pore size distribution upon Zn incorporation (Fig. 5(j–l)) suggest partial pore occupation or structural rearrangement due to metal coordination. Zn@COF-1 and Zn@COF-2 retain significant porosity, ensuring efficient gas transport, while Zn@COF-3 shows a slight reduction in pore size, which might enhance adsorption and charge transfer efficiency for NH_3_ sensing. High-resolution TEM analysis of Zn@COF-3 (Fig. 6(a–c)) reveals well-defined lattice fringes with a spacing of 0.31 nm, corresponding to the *d*_(110)_ value of Zn@COF-3, which enhances electronic conductivity and sensing response. The FFT pattern (Fig. 6(d)) confirms the crystalline nature, which facilitates efficient electron transfer upon NH_3_ interaction. Additionally, elemental mapping (Fig. 6(e–i)) and EDAX images Fig. S2 (ESI†) confirm the homogenous distribution of C, N, O, and Zn, ensuring uniform active sites for NH_3_ adsorption and charge transfer. The porous and fibrous network of Zn@COF-1 and Zn@COF-2 facilitates rapid gas diffusion, while the micro-flower-like structure of Zn@COF-3 provides a large surface area for enhanced NH_3_ adsorption and improved charge transfer efficiency. Furthermore, the high crystallinity and well-structured Zn sites improve conductivity and selectivity, making Zn@COFs more effective NH_3_ sensing materials than the pristine COFs.

#### 3.1.5 Atomic force microscopy (AFM) analysis

Similarly, Fig. S3(a–c) (ESI†) presents the atomic force microscopy (AFM) surface morphology of the Zn@COF samples with magnifications of 10 μm^2^ × 10 μm^2^. The AFM contact mode was employed to obtain the area roughness parameters of the MCOFs. The mean surface roughness (*S*_a_) for Zn@COF-1 is 260.16 nm, that of Zn@COF-2 is 292.33 nm, and that of Zn@COF-3 is 471.75 nm. The root mean square (*S*_q_) values of the Zn@COF-1, 2 and 3 samples are 562.36 nm, 671.06 nm, and 903.45 nm, respectively. High surface roughness was found in the Zn@COF-3 sample; this roughness of the films provides effective surface area, adsorption sites, and gas reactivity.^71^

#### 3.1.6 X-ray photoelectron spectroscopy (XPS)

**Fig. 7:**
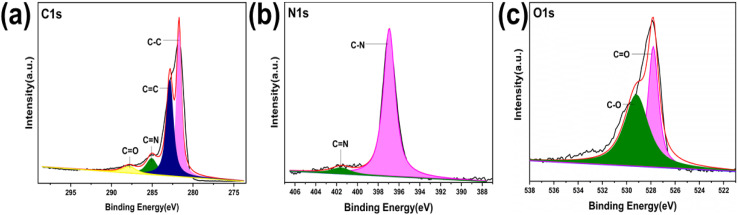
High-resolution XPS spectra of COF-3: (a) carbon atom, (b) nitrogen atom, and (c) oxygen atom.

**Fig. 8:**
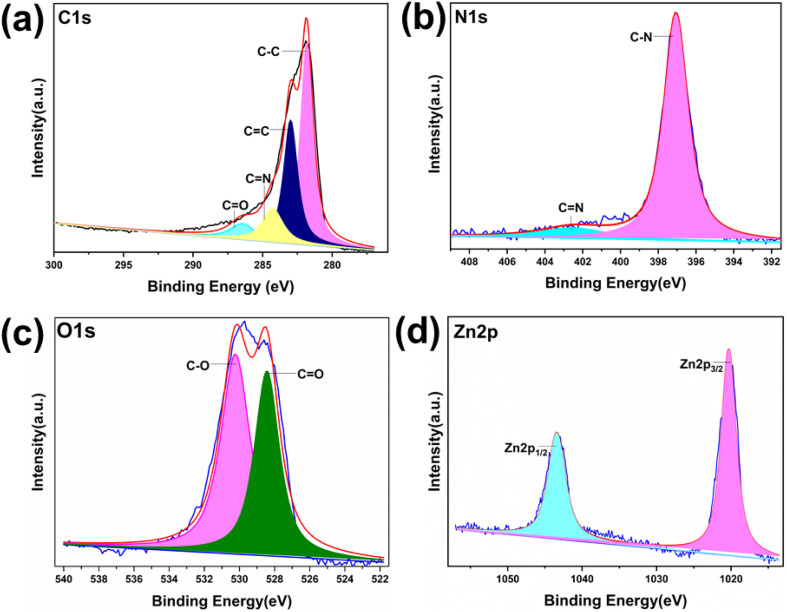
High-resolution XPS spectra of Zn@COF-3: (a) carbon atom, (b) nitrogen atom, (c) oxygen atom, and (d) Zn atom.

Analysis of the XPS survey spectra confirmed the presence of C, O, N, and Zn (Fig. S4, ESI†). The C 1s spectra with high resolution reveal the existence of four distinct carbon species on the surface of the samples: C–C, C=N, C=C, and C=O (Fig. 7(a), 8(a), and Fig. S5–S8, ESI†). The N 1s peak of COF-1 shifted from 399.44 eV to 399.2 eV in Zn@COF-1 (Fig. S7(b) ESI†), indicating the successful coordination between Zn^2+^ and the N atom. In COF-2 and COF-3, similar coordination was observed between Zn^2+^ and N atoms (Fig. 8(b) and S8(b), ESI†).^85^ COF-1 exhibits energy peaks at 528.0 eV and 529.8 eV in its high-resolution O 1s XPS spectrum (Fig. S5(c), ESI†). COF-2 exhibits energy peaks at 528.4 eV and 530.4 eV (Fig. S6(c), ESI†). Similarly, COF-3 exhibits peaks at 527.84 eV and 530.5 eV (Fig. 7(c)).^86,87^ The Zn2p spectra in Fig. 8(d), S7(d) and S8(d) (ESI†) for Zn@COF-1, 2, and 3, respectively, show two distinct peaks at 1042.8 eV and 1019.9 eV. The binding energy for Zn 2p_3/2_ is 1042 eV, while the binding energy for Zn 2p_1/2_ is 1019 eV.^88^ Following the treatment of COF-1 with Zn^2+^, a new Zn–O peak at 528.3 eV emerged, and the COF-1 peak at 528.6 eV was reduced to a certain degree, indicating that a portion of the oxygen atoms of C=O were coordinated with Zn^2+^. A new peak corresponding to Zn–O bonding was seen in the cases of COF-2 and COF-3 treated with Zn^2+^. The C=O bonding in COF-2 and COF-3 corresponded to the peaks at 528.4 eV and 527.84 eV, respectively. These peaks show decreased intensity in Zn@COF-1, 2 and 3. This may be due to the coordination of Zn^2+^ with the oxygen atoms of the C=O groups of COFs.^89^ This confirms the successful incorporation of zinc into the COF framework, as well as the characteristic zinc peaks and variations in binding energies.

#### 3.1.7 Brunauer–Emmett–Teller (BET) analysis

The N_2_ adsorption–desorption isotherms of COF-1, COF-2, and COF-3 (Fig. S9(a–c), ESI†) exhibit type-IV hysteresis loops, which are indicative of mesoporous frameworks with well-defined pore channels. Similarly, Zn@COF-1, Zn@COF-2, and Zn@COF-3 (Fig. S9(d–f), ESI†) also showed type-IV behavior, confirming that the mesoporous nature of the parent COFs was retained after Zn^2+^ incorporation. Notably, a significant enhancement in BET surface area was observed upon zinc doping: COF-1, COF-2, and COF-3 exhibited surface areas of 122 m^2^ g, 191 m^2^ g, and 253 m^2^ g, respectively (Table 1), whereas Zn@COF-1, Zn@COF-2, and Zn@COF-3 showed increased values of 273 m^2^ g^−1^, 320 m^2^ g^−1^, and 335 m^2^ g^−1^, respectively (Table 1). This increase in surface area after zinc incorporation is likely due to improved framework ordering and pore accessibility, which facilitate enhanced gas interactions. These findings indicate that all the Zn@COF materials can adsorb, making them effective gas sensors.^90–92^

**Table 1:** BET surface area values of pristine COFs and Zn@COFs

S. no.	Material	Surface area
1	COF-1	122 m^2^ g^−1^
2	COF-2	191 m^2^ g^−1^
3	COF-3	253 m^2^ g^−1^
4	Zn@COF-1	273 m^2^ g^−1^
5	Zn@COF-2	320 m^2^ g^−1^
6	Zn@COF-3	335 m^2^ g^−1^

#### 3.1.8 Thermogravimetric analysis (TGA)

Thermogravimetric analysis (TGA) revealed that the COFs and MCOFs possess good thermal stability up to 350–400 °C, as indicated by the less than 10% weight loss when the temperature was increased from room temperature to 350 °C·As shown in Fig. S10(a–c) (ESI†) the TGA graphs of all the COFs and MCOFs gave pyrolysis temperatures in the approximate range of 350 °C to 450 °C with approximately 35% pyrolysis residue.^93^

### 3.2 Gas sensing performance

Ammonia gas sensing investigations at ambient temperature using Schiff-based COFs and MCOFs have not been reported, although they have been provided in NO_2_ and H_2_ sensing research in the literature.^94,95^ The presence of keto–enol tautomerism in the present COFs and MCOFs significantly enhances the gas sensing performance owing to their structural flexibility and dynamic behaviour. The ability to switch between keto and enol forms can create different binding sites, electronic states, and the potential to alter the charge distribution within the COFs, which is useful for enhancing the gas sensing and selectivity of ammonia gas.^96^

Therefore, in the current investigation, a quantitative-based sensor for ammonia, acetic acid, formaldehyde, and ethanol gas detection was fabricated with the COFs and MCOFs at room temperature. The current study focused on four representative gas analytes, namely, ammonia, formaldehyde, ethanol, and acetic acid, at concentrations of 1, 10, 25, and 50 ppm to demonstrate the chemiresistive gas sensing capability of the parent COFs (COF-1, 2, and 3) and the MCOFs (Zn@COF-1, 2 and 3).

#### 3.2.1 Gas sensing performance of parent COFs (COF-1, 2, and 3)

The COF-1 materials demonstrate gas-sensing responses of 1.02, 1.63, 4.45, and 11.65 to ammonia at concentrations of 1, 10, 25, and 50 ppm, respectively. For ethyl alcohol, the corresponding responses are 1.12, 1.50, 1.63, and 1.89. The response times for ammonia at concentrations of 1, 10, 25, and 50 ppm are 118 s, 175 s, 163 s, and 116 s, respectively. The recovery times for ammonia are 82 s, 64 s, 58 s, and 48 s. For ethyl alcohol, the response times are 42 s, 59 s, 58 s, and 48 s, with recovery times of 34 s, 37 s, 38 s, and 36 s. Additionally, this compound does not detect acetic acid or formaldehyde gas at ambient temperatures; these values are summarized in Table S3 (ESI†). Similarly, the COF-2 materials demonstrate gas-sensing responses of 1.2, 9.8, 14.14, and 23.88 for ammonia at concentrations 1, 10, 25, and 50 ppm, respectively. For acetic acid, the responses are 1.35, 4.2, 8.1, and 17.24. The response times for ammonia at different concentrations (1, 10, 25, and 50 ppm) are 110 s, 177 s, 136 s, and 79 s, respectively. The recovery times for ammonia are 40 s, 61 s, 49 s, and 46 s. For acetic acid, the response times are 30 s, 37 s, 43 s, and 41 s with recovery times of 23 s, 69 s, 56 s, and 51 s. Additionally, this compound does not detect ethyl alcohol or formaldehyde gas at ambient temperature; these values are summarized in Table S3 (ESI†).

The COF-3 materials demonstrated gas-sensing responses of 1.5, 10.5, 21.6, and 55.8 for ammonia at different concentrations of 1, 10, 25, and 50 ppm, respectively. For acetic acid, the response values were 1.34, 4.08, 7.64, and 15.80 at the same concentrations. It showed 1.2, 1.26, 1.29, and 1.47 responses to ethyl alcohol, while those for formaldehyde were 1.13, 1.16, 1.19, and 1.26. The response times of COF-3 to ammonia at concentrations of 1, 10, 25, and 50 ppm were 38 s, 43 s, 39 s, and 34 s respectively, while the recovery times for these concentrations were 22 s, 36 s, 33 s, and 29 s. Similarly, the response times were 36 s, 54 s, 30 s, and 27 s, and the recovery times 23 s, 29 s, 13 s, and 15 s for acetic acid. The response values and graphs of ammonia, acetic acid, ethyl alcohol, and formaldehyde gas are shown in Fig. S11(a–d) and Table S3 (ESI†). Comparing the gas sensing performance of the COFs, COF-3 detects all gases and shows superior responses, response times, and recovery times than COF-1 and COF-2. COF-1 could not detect the gases acetic acid and formaldehyde, while COF-2 failed to detect ethyl alcohol and formaldehyde. The enhanced sensing activity of COF-3 is attributed to its highly crystalline nature, spongy flower morphology, and an increase in keto–enol active sites.

#### 3.2.2 Gas sensing performance of Zn@COFs

Similarly, the response values of the Zn@COF-1 material to ammonia at concentrations of 1, 10, 25, and 50 ppm are 1.65, 2.83, 5.54, and 20.24, respectively; the response times are 105 s, 163 s, 121 s, and 53 s, with recovery times of 31 s, 24 s, 19 s, and 28 s. For acetic acid, the response values were 1.24, 1.58, 5.1, and 10; the response times were 37 s, 39 s, 65 s, and 60 s, with recovery times of 28 s, 36 s, 75 s, and 43 s. Ethyl alcohol gave response values of 1.42, 1.58, 1.69, and 1.76; the response times were 30 s, 39 s, 41 s, and 57 s with recovery times of 24 s, 26 s, 29 s, and 40 s. For formaldehyde, the response values were 1.13, 1.37, 1.45, and 1.59, and the response times were 17 s, 37 s, 41 s, and 49 s with recovery times of 13 s, 14 s, 26 s, and 29 s. The response graphs for ammonia, acetic acid, ethyl alcohol, and formaldehyde gas are presented in Fig. S12(a–d) and Table S4 (ESI†).

The response values of the Zn@COF-2 material for ammonia at different concentrations of 1, 10, 25, and 50 ppm were 1.65, 11.83, 23.18, 72.85, and the response times were 100 s, 167 s, 125 s, and 66 s with recovery times of 29 s, 34 s, 31 s, and 41 s, respectively. For acetic acid, the response values were 1.44, 4.34, 8.84, and 10; the response times were 26 s, 38 s, 39 s, and 40 s with recovery times of 17 s, 65 s, 55 s, and 62 s. For ethyl alcohol, the response values were 1.46, 2.87, 3.21, and 4.78, and the response times were 30 s, 43 s, 45 s, and 58 s with recovery times of 23 s, 32 s, 34 s, and 42 s. For formaldehyde, the response values were 1.13, 1.36, 1.45, and 1.56; the response times were 18 s, 31 s, 22 s, and 30 s with recovery times of 17 s, 25 s, 30 s, and 22 s, and the response values and graphs for ammonia, acetic acid, ethyl alcohol, and formaldehyde gas are presented in Fig. S13(a–d) and Table S4 (ESI†).

**Fig. 9:**
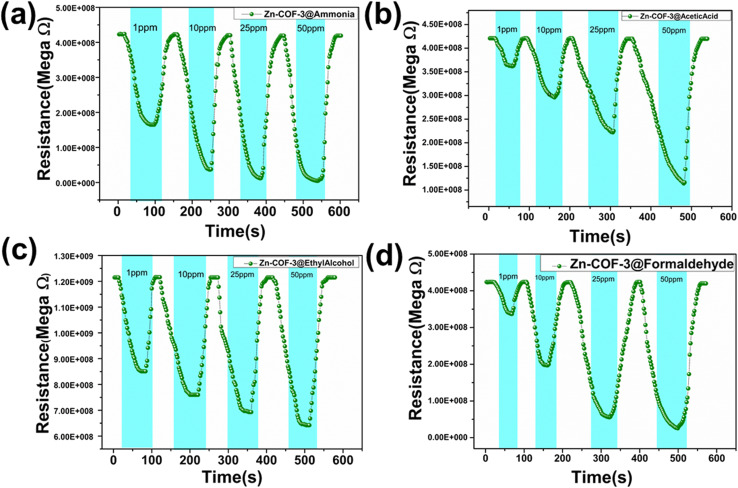
Resistance curves of Zn@COF-3 at concentrations of 1, 10, 25, and 50 ppm for: (a) ammonia, (b) acetic acid, (c) ethyl alcohol, and (d) formaldehyde.

The Zn@COF-3 material exhibited response values of 2.54, 11.58, 36.13, and 94.90 at ammonia concentrations of 1, 10, 25, and 50 ppm; the response times were 26 s, 28 s, 25 s, and 17 s with recovery times of 18 s, 12 s, 8 s, and 6 s, showing enhanced results compared to the other COFs and Zn@COFs. For acetic acid, the response values were 1.54, 4.28, 8.54, and 17.80; the response times were 32 s, 46 s, 27 s, and 23 s with recovery times of 17 s, 22 s, 10 s, and 8 s. For ethyl alcohol, the response values were 1.42, 1.47, 1.57, and 1.9, and response times were 42 s, 57 s, 48 s, and 50 s with recovery times of 12 s, 24 s, 24 s, and 31 s. For formaldehyde, the response values were 1.11, 1.23, 1.28, and 1.36; the response times were 25 s, 41 s, 20 s, and 39 s with recovery times 21 s, 24 s, 53 s, and 31 s. The response values and graphs for ammonia, acetic acid, ethyl alcohol, and formaldehyde gas are presented in Fig. 9(a–d) and Table S4 (ESI†).

**Fig. 10:**
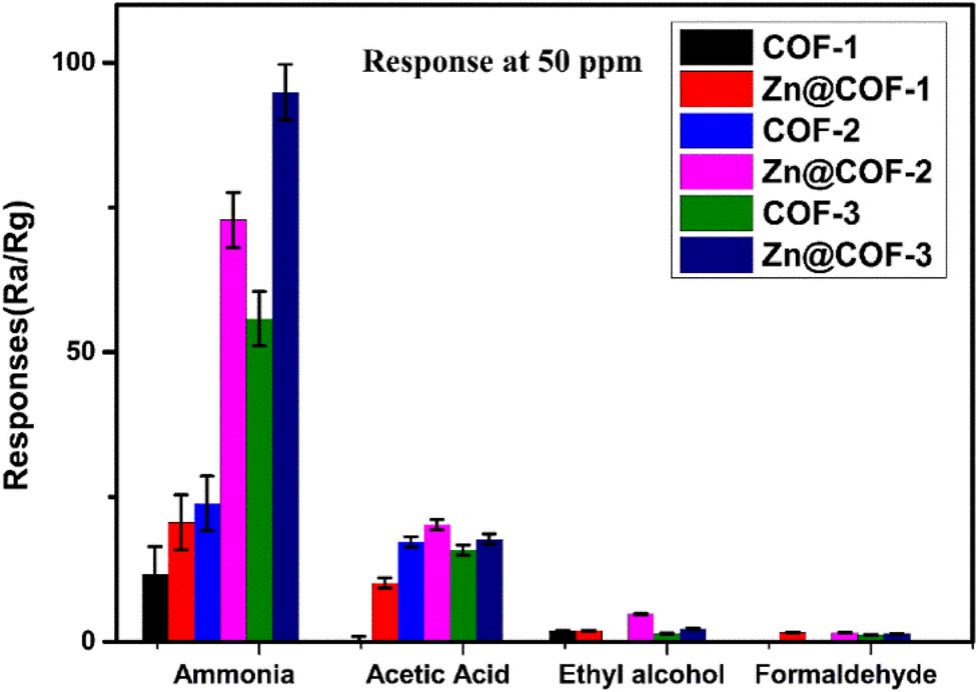
Sensing comparison of ammonia, acetic acid, ethanol, and formaldehyde using the parent COFs (COF-1, 2 and 3) and MCOFs (Zn@COF-1, 2 and 3).

Comparing the gas sensing performance of COFs and MCOFs, the MCOFs demonstrate superior results. The response time and recovery time of the COFs at 1 ppm were as follows: COF-1 (*t*_res_ = 118 s, *t*_rec_ = 42 s at 1 ppm), COF-2 (*t*_res_ = 110 s, *t*_rec_ = 40 s at 1 ppm), and COF-3 (*t*_res_ = 38 s, *t*_rec_ = 22 s at 1 ppm). Similarly, the response times and recovery times of the MCOFs at 1 ppm were as follows: Zn@COF-1 (*t*_res_ = 105 s, *t*_rec_ = 31 s at 1 ppm), Zn@COF-2 (*t*_res_ = 100 s, *t*_rec_ = 29 s at 1 ppm), and Zn@COF-3 (*t*_res_ = 26 s, *t*_rec_ = 18 s at 1 ppm). Fig. 10 and Table 2 present a comparison of the ammonia sensor responses with those of previous reported sensors. The zinc-doped COFs (Zn@COF-3) show superior detection responses and recovery times due to the incorporation of zinc metal, which increases the active sites and enhances the interaction with ammonia gas. Additionally, it enhances the stability, selectivity, electrical conductivity, surface area, and response times, making Zn@COFs superior for NH_3_ sensing compared to traditional COFs. Comparing the MCOFs, Zn@COF-3 shows superior results to Zn@COF-1 and 2, which this may be attributed to Zn@COF-3 having abundant active sites, keto–enol tautomerism, high surface area, and pore volume, which have a substantial impact on gas sensors.^109–112^

**Table 2:** Comparison of ammonia sensing performance of the present sensor with previously reported studies at ambient temperature^a^

Sensor	LOD	*T*_res_	*T*_rec_	*T* °C	Ref.
ZnCo(NA)	1 ppm	39 s	40 s	RT	97
ZnO/SiO_2_	10 ppm	65 s	60 s	RT	98
TiO_2_	5 ppm	34 s	90 s	RT	99
ZnO/Pd	30 ppm	198 s	334 s	RT	100
Cu-BTC@GO	500 ppm	20 s	30 s	RT	101
CuHITP_2_	100 ppm	1.7 s	3.34 s	RT	102
CuPc@IRMOF-3	0–50 ppm	NA	NA	RT	103
COF-DC-8	NA	NA	NA	RT	104
pCTF	100 ppm	100 s	400 s	RT	105
TAPB-BPDA COF	100 ppm	8–40 s	100–120 s	RT	106
CTF-1-A	100 ppm	100 s	420 s	NA	107
HMP-TAPB-1	500 ppm	65 s	100–9 s	NA	108
COF-1	1 ppm	118 s	42 s	RT	This work
COF-2	1 ppm	110 s	40 s	RT	This work
COF-3	1 ppm	38 s	22 s	RT	This work
Zn@COF-1	1 ppm	105 s	31 s	RT	This work
Zn@COF-2	1 ppm	100 s	29 s	RT	This work
Zn@COF-3	1 ppm	26 s	18 s	RT	This work

a LOD – limit of detection, *T*_res_ – response time, *T*_rec_ – recovery time, *T* °C – temperature, and Ref. – references.

**Fig. 11:**
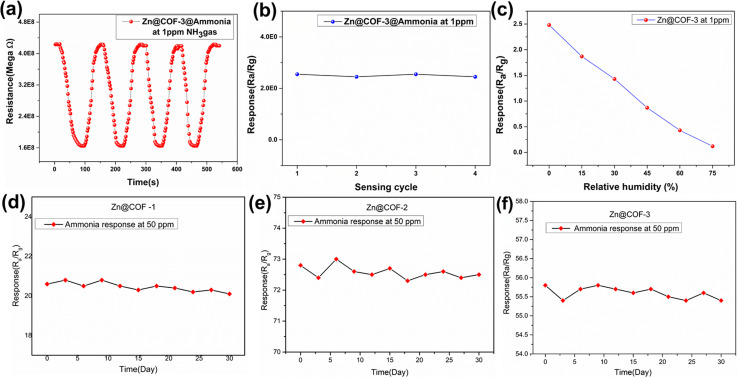
(a and b) Reproducibility of Zn@COF-3 at 1 ppm. (c) Response (*R*_a_/*R*_g_) of the Zn@COF-3 sensor to 1 ppm of ammonia gas under varying relative humidity (RH) conditions. (d–f) Long-term stability of the responses of Zn@COF-1, Zn@COF-2, and Zn@COF-3 towards ammonia gas at 50 ppm.

One of the most important aspects of sensing materials is their long-term stability, reproducibility, and relative humidity sensing. Fig. 11(a and b) presents a reproducibility analysis of the consistent resistance variations and stable response across four cycles, confirming the reliability of Zn@COF-3 at 1 ppm of NH_3_. Fig. 11(c) reveals a decreasing response with increasing relative humidity, suggesting that water molecule adsorption competes with ammonia adsorption, leading to reduced sensitivity. However, the Zn@COF-3 sensor maintains a detectable response, indicating its practical applicability. The term stability refers to the extent to which a sensor's properties remain consistent over time. To evaluate the performance of the Zn@COFs as sensing materials, their stability and repeatability were assessed over 30 days at a concentration of 50 parts per million (ppm) as shown in Fig. 11 (d–f), which present the stability response graphs over the entire 30-day period for the three Zn@COFs, confirming their stability.

### 3.3 Gas sensing mechanism

The gas sensors exhibited n-type sensing characteristics, rendering them less resistant to reducing gases such as ammonia, acetic acid, ethyl alcohol, and formaldehyde due to the surface-related chemical reactions of the target gas molecules. The sensing materials performance is significantly affected by their composition, specific surface area, and valence states. The sensing mechanism of the MCOFs is the creation of a charge depletion layer on the MCOF surface as a result of electron trapping on adsorbed oxygen species.^109^ The overall gas sensing process includes gas adsorption, surface reactions, and desorption processes. The chemosensor undergoes a process in which oxygen molecules attach to its surface. This results in the creation of more active ionized oxygen species (O and O_2_^−^) and the formation of holes as they take electrons from the Schiff-based Zn@COF-1, 2, and 3 (eqn (3) and (4)). Due to the capture of conduction band electrons and the expansion of the surface electron depletion layer in Zn@COF-1, 2 and 3, the conductance of the sensor decreases, leading to an increase in resistance.^113–115^

3 O_2(gas)_ → O_2(ads)_

4 O_2(ads)_ + e^−^ → O_2(ads)_^−^

5 O_2(ads)_^−^ + e^−^ → 2O_(ads)_^−^

6 O_(ads)_^−^ + e^−^ → O_2(ads)_^−^

**Fig. 12:**
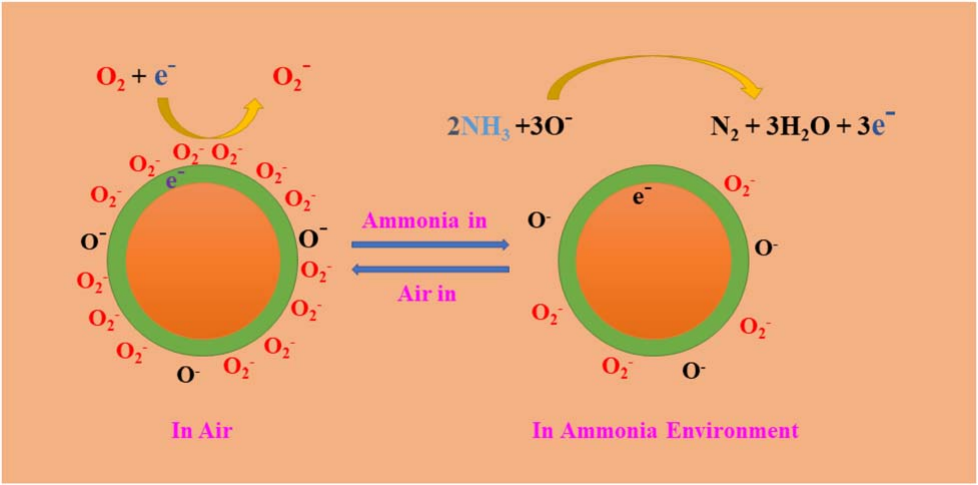
Mechanism diagrams of 2D-MCOF sensors in the air and targeted gas.

When the resistance of the sensor decreases, trapped electrons are released back into the conduction band. This happens when the sensor is exposed to target gases such as ammonia and interacts with adsorbed oxygen ions (Fig. 12). The above equations (eqn (5) and (6)) provide a brief overview of the oxygen adsorption depletion layer and subsequent reactions for ammonia sensing, and the subsequent equations (eqn (7)–(10)) involve the air and targeted gases.

7 NH_3_ + O_2(ads)_ → N_2_ + H_2_O (ammonia)

8 HCHO + O_2(ads)_^−^ → CO_2_ + H_2_O + e^−^ (formaldehyde)

9 CH_3_CH_2_OH + 6O_(ads)_^−^ → 2CO_2_(g) + 3H_2_O(g) + 6e^−^ (ethyl alcohol)

10 CH_3_COOH + 4O_(ads)_^−^ → 2CO_2_(g) + 2H_2_O(g) + 4e^−^ (acetic acid)

**Fig. 13:**
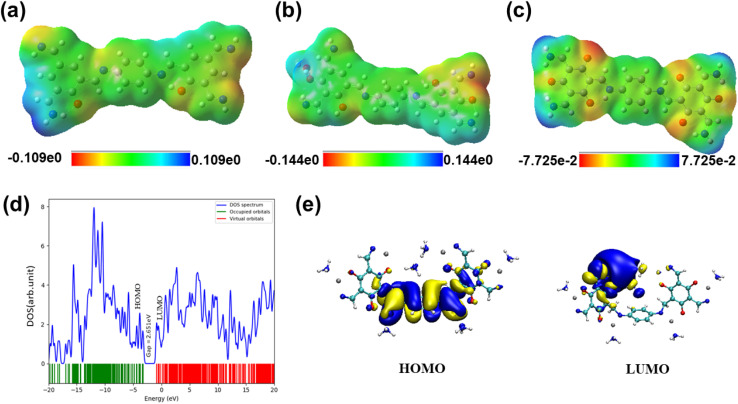
Adsorption energies for (a) Zn@COF-1@NH_3_, (b) Zn@COF-2@NH_3_, and (c) Zn@COF-3@NH_3_. (d) Density of states for Zn@COF-3, and (e) HOMO–LUMO plots of Zn@COF-3.

Further, to support the above mechanism, DFT simulation studies were performed. The electrostatic distributions of Zn@COF and NH_3_ show potential adsorption areas where NH_3_ might interact with Zn^2+^. The study also simulated potential interactions between NH_3_ gas molecules and the surfaces of Zn@COF-1, 2, and 3. Fig. 13(a–c) shows the original and geometrically optimised arrangements of NH_3_ on these surfaces, while Fig. S16(a–c) (ESI†) illustrates the optimised arrangements of Zn@COF-1, 2, and 3. It was also observed that the Zn^2+^ interlayer functions as a trap site for NH_3_ gas molecules. The binding energy of Zn@COF-3 is −281.77 kJ mol^−1^, which is higher than that of Zn@COF-1 (−56.51 kJ mol^−1^) and Zn@COF-2 (−171.1 kJ mol^−1^). These findings suggest that Zn@COF-3 has a stronger affinity for NH_3_ due to its extensive surface area. The HOMO is localised on the phenylenediamine unit (donor) and LUMO on the 1,3,5-hydroxybenzene-2,4,6-tricarbaldehyde unit (acceptor) (Fig. 13(e), S17(a) and (b) (ESI†)); the band gap energy values are listed in Table S5 (ESI†). The gas adsorption energy values indicated by the density of states (DOS) graphs are presented in Fig. 13(d), S18(a), (b) and Table S6 (ESI†).

## 4. Conclusion

In this study, we successfully synthesized COFs and Zn-encapsulated COFs (Zn@COFs) for highly efficient NH_3_ sensing at room temperature. The incorporation of Zn^2+^ into the COF framework was confirmed by ^13^C CP-MAS NMR spectroscopy (C=O, peak at ∼183 ppm, and imine C=N peaks at ∼148 and ∼146 ppm) and XPS (C=O peak at 527.84 eV, C=N at 399.2 eV, Zn 2p_3/2_ peak at 1042 eV, and Zn 2p_1/2_ at 1019 eV). Among the synthesized materials, Zn@COF-3 exhibited superior NH_3_ sensing performance, with a low detection limit of 1 ppm, a rapid response time of 26 s, and a fast recovery time of 18 s. These remarkable properties can be attributed to its micro-flower-like morphology, high crystallinity, and large surface area (335 m^2^ g^−1^), which facilitate efficient gas interaction and charge transfer. DFT calculations further confirmed a strong interaction between NH_3_ molecules and Zn@COF-3, with a high adsorption energy of −281.77 kJ mol^−1^ and a low energy gap of 2.651 eV, leading to enhanced charge transfer efficiency. These characteristics make the Zn@COFs significantly more effective than the pristine COFs for NH_3_ sensing at room temperature. Given their exceptional sensing properties, Zn@COFs hold great potential for applications in environmental monitoring, industrial safety, and medical diagnostics. Future research can explore their integration into flexible and wearable sensors, portable miniaturized devices, multi-gas sensing applications, and IoT-based environmental monitoring systems.

## Data availability

The data that supports the findings of this study are available in the ESI† of this article.

## Author contributions

Sujith Benarzee Nallamalla – experimental, methodology, data acquisition, formal analysis, and writing – original draft. Naresh Kumar Katari – visualisation, data curation, and resources. A. Jagan Mohan Reddy – data curation, Sreekantha Babu Jonnalagadda – formal analysis and data curation. Surendra Babu Manabolu Surya – conceptualisation, methodology, supervision, review and editing.

## Conflicts of interest

The authors report no declarations of interest.

## Supplementary Material

RA-015-D5RA01430A-s001

RA-015-D5RA01430A-s002

RA-015-D5RA01430A-s003

RA-015-D5RA01430A-s004
