# Supplementary material for: Zinc-encapsulating covalent organic frameworks for enhanced chemiresistive NH3 sensing at room temperature
Source: RSC Adv. 2025 May 19;15(21):16708–23. doi: 10.1039/d5ra01430a (PMC12087901; doi:10.1039/d5ra01430a)
Supplement: RA-015-D5RA01430A-s004 [file RA-015-D5RA01430A-s004.pdf]

Supplementary Information (SI) for RSC Advances.
This journal is © The Royal Society of Chemistry 2025

Electronic Supplementary Information for RSC Advances

Zinc encapsulate - Covalent Organic Frameworks for enhanced chemiresistive NH_3 sensing at room temperature

Sujith Benarzee Nallamalla[a], Naresh Kumar Katari[b], A. Jagan Mohan Reddy[c], Sreekantha Babu Jonnalagadda[b] , Surendra Babu Manabolu Surya[a*]

[a] *Department of Chemistry, GITAM University, Hyderabad - 502329, Telangana, India.*
[b] *School of Chemistry & Physics, College of Agriculture, Engineering & Science, Westville Campus, University of KwaZulu-Natal, P Bag X 54001, Durban 4000, South Africa.*
[c] *Departments of Chemistry, CMR Technical Campus, Medchal, Hyderabad 501401, India.*

***Correspondence:** smanabol@gitam.edu (M. S. Surendra Babu)

Table of Contents

Section	Contents	Page No
S-1	Section S-1: Chemicals and Reagents	3
S-2	Section S-2: General Information	3
S-3	Section S-3:^{13}C CP-MAS solid-state NMR spectra	4
S-4	Section S-4: Energy Dispersive X-ray Analysis	5
S-5	Section S-5: Atomic Force Microscopy	5
S-6	Section S-6: X-ray Photoelectron Spectroscopy	6-8
S-7	Section S-7: Brunauer–Emmett–Teller (BET) analysis	9
S-8	Section S-8: Thermogravimetric Analysis (TGA)	10
S-9	Section S-9: Gas Sensing Responses Graphs	11-13
S-10	Section S-10: Theoretical Studies and Results	14-15
S-11	Section S-11: Tables	16-34

Section S-1: Chemicals and Reagents

Chemicals and Reagents: The materials, Phloroglucinol (98%, AVRA Synthesis Ltd., Telangana, India), benzene-1,4-diamine (99%, Sigma Aldrich), Mesitylene (99%, AVRA Synthesis Ltd., Telangana, India) and 1,4 Dioxane (99%, SDFCL., Mumbai, India), Acetic acid (99.8% BLD Pharma Ltd., Telangana, India), and Zinc Chloride (98%, Sd fine-Chem Ltd., Mumbai, India), Hexamethylenetetramine (99%, Sigma Aldrich), Tri fluoro acetic acid(99%, AVRA Synthesis Ltd., Telangana, India) acetone, hexane, and anhydrous THF(99.8% BLD Pharma Ltd., Telangana, India).

Section S-2: General Information

General Information: X-ray diffraction (XRD) was performed by using Rigaku Ultima IV powder XRD by Cu Kα radiation (15.4 nm) at 40 kV, 30 mA, and 2 deg/ min, and the scanning range was 5–70°. FTIR spectra are recorded with the Agilent ATR benchtop spectrometer 4500-400 cm-1 using KBr. Solid-state ^{13}C CP-MAS (Cross-Polarization with Magic Angle Spinning) spectra were recorded on a JEOL (model ECX-400 MHz) spectrometer to confirm synthesized MCOFs. A scanning electron microscope (ZEIS Evo SEM, EDAX Oxford instruments) was used to analyze the morphology and particle size. Transmission electron microscopy (TEM) was performed using Talos F200 S (Thermo Fischer) operating at 200 kV. Quanta Chrome and NOVA Win conducted a study on the adsorption-desorption of nitrogen and examined surface area, pore size, and pore volume. The elemental electronic state of the adsorbent surface was confirmed by X-ray photoelectron spectroscopy (XPS) analysis of the composite using the AXIS Supra- Shimadzu model, Japan, and Atomic force microscopy (AFM) is carried out using Agilent 5500 in tapping mode. TGA analyses were done on a Netzsch TG209F1 apparatus at 10 K min-1 under N_2 atmosphere.

Section S-3: ^{13}C CP-MAS solid-state NMR spectra

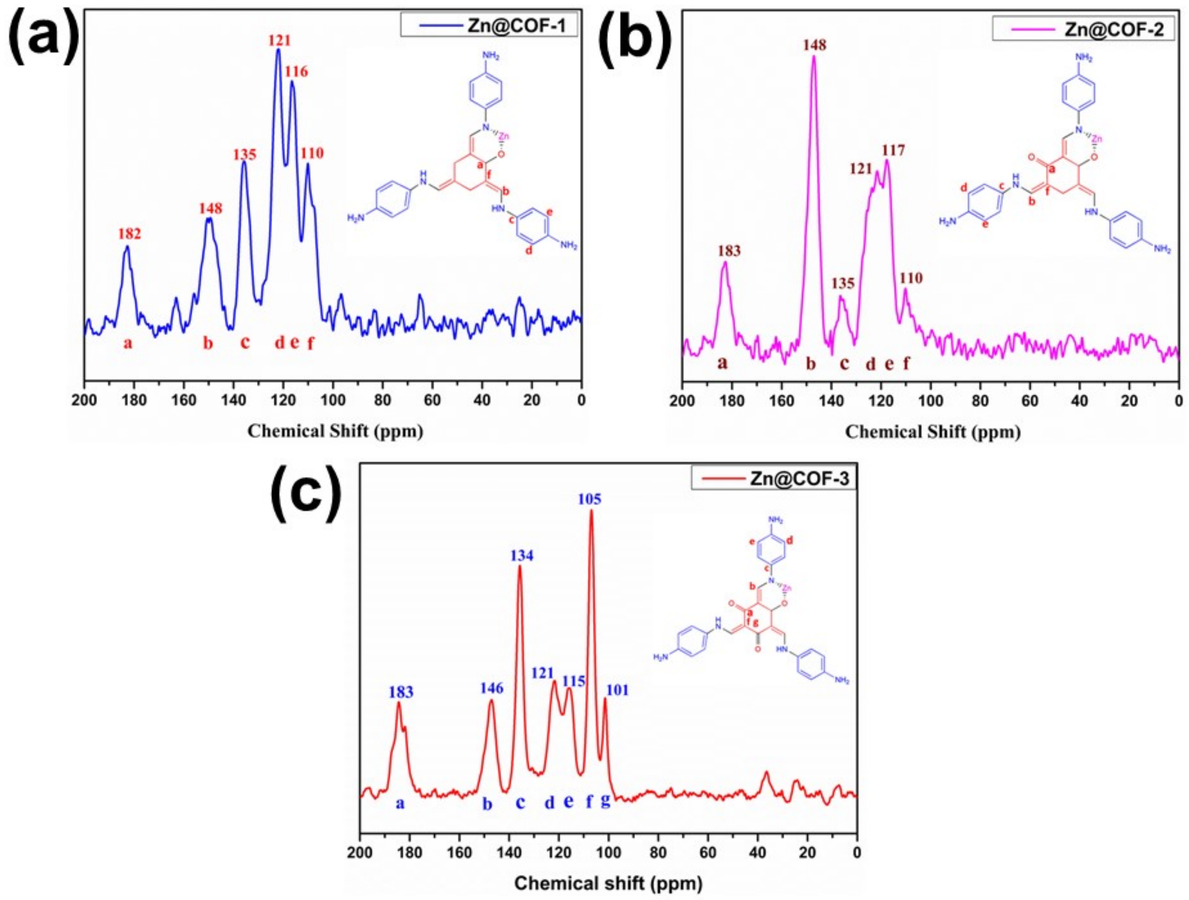

Fig. S1. ^{13}C CP-MAS solid-state NMR spectra of (a) Zn@COF-1, (b) Zn@COF-2, and (3) Zn@ COF-3.

Section S-4: Energy Dispersive X-ray Analysis

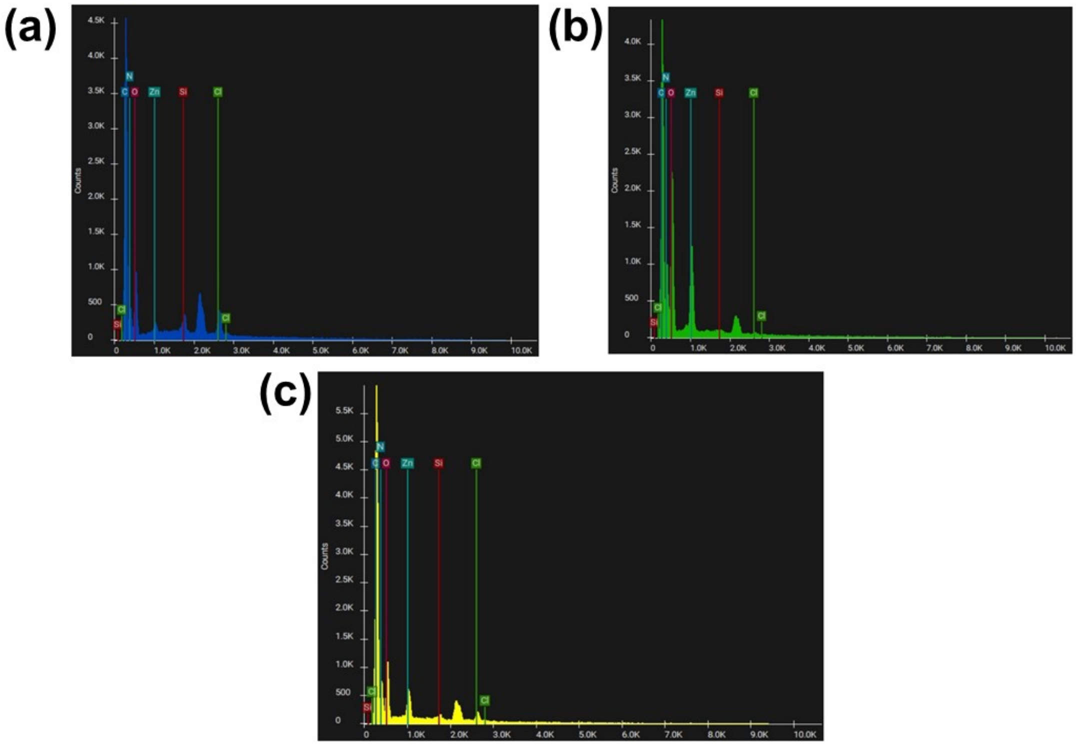

Fig. S2. EDX images of (a) Zn@COF-1, (b) Zn@COF-2, and (c) Zn@COF-3

Section S-5: Atomic Force Microscopy

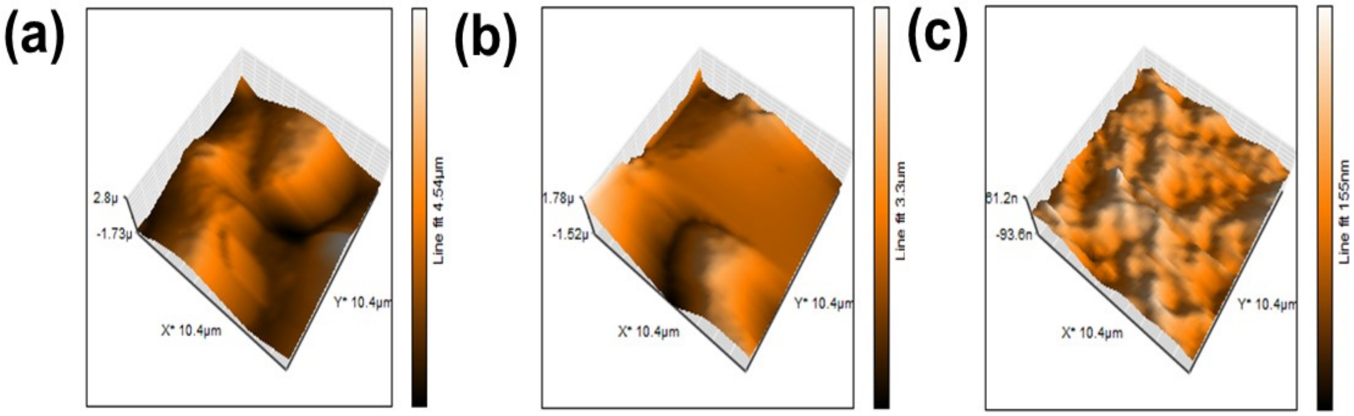

Fig. S3. AFM 3D images of (a) Zn@COF-1, (b) Zn@COF-2 and (c) Zn@COF-3.

Section S-6: X-ray Photoelectron Spectroscopy

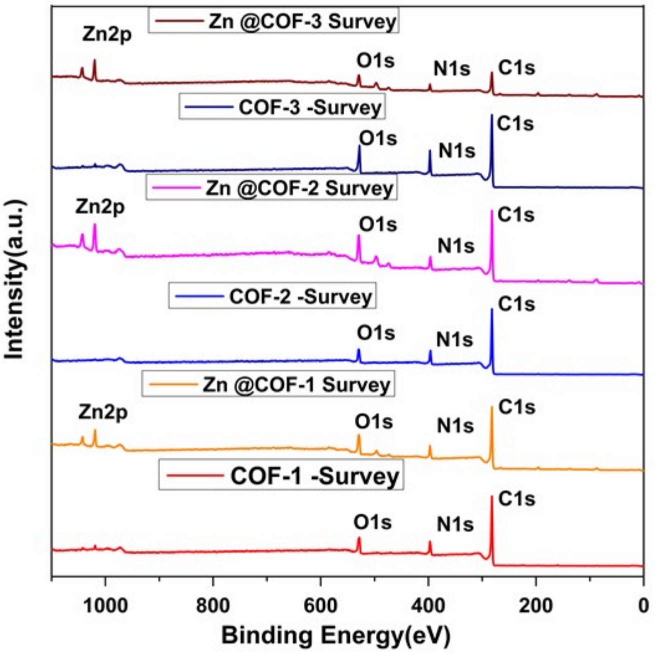

Fig. S4. High-resolution XPS Spectra Survey graphs of all Synthesised COFs and MCOFs.

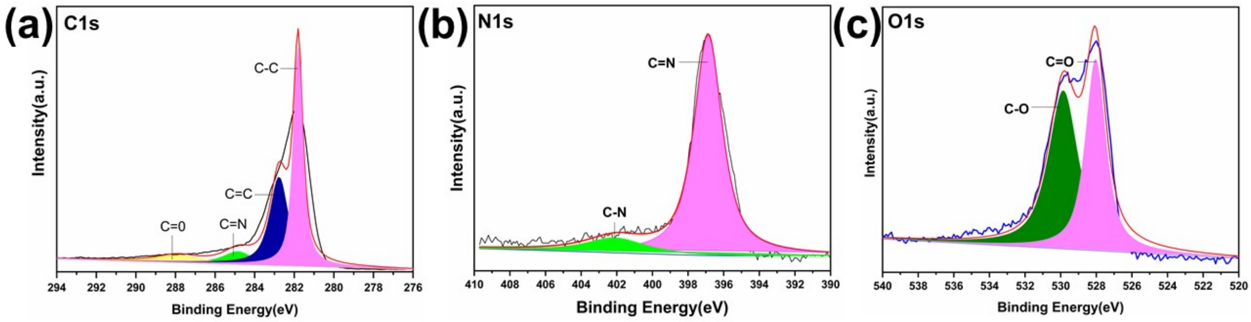

Fig. S5. High-resolution XPS spectra of COF-1 (a) Carbon atom, (b) Nitrogen atom, and (c) oxygen atom.

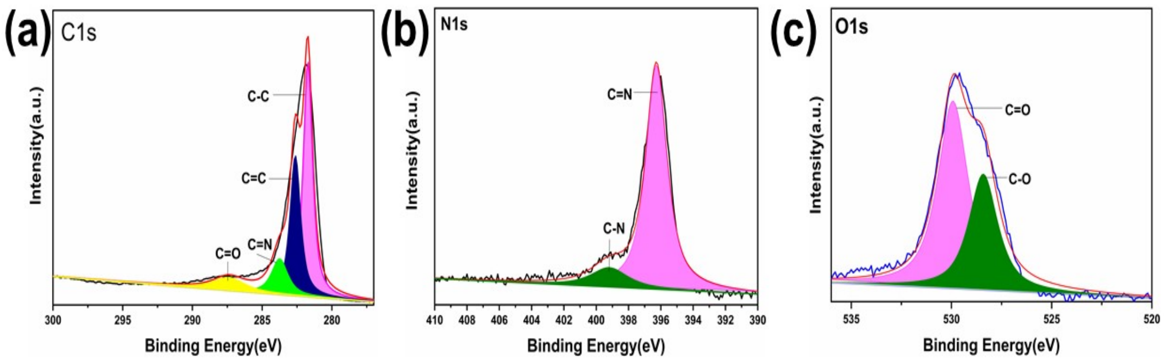

Fig. S6. High-resolution XPS spectra of COF-2 (a) Carbon atom, (b) Nitrogen atom, and (c) oxygen atom.

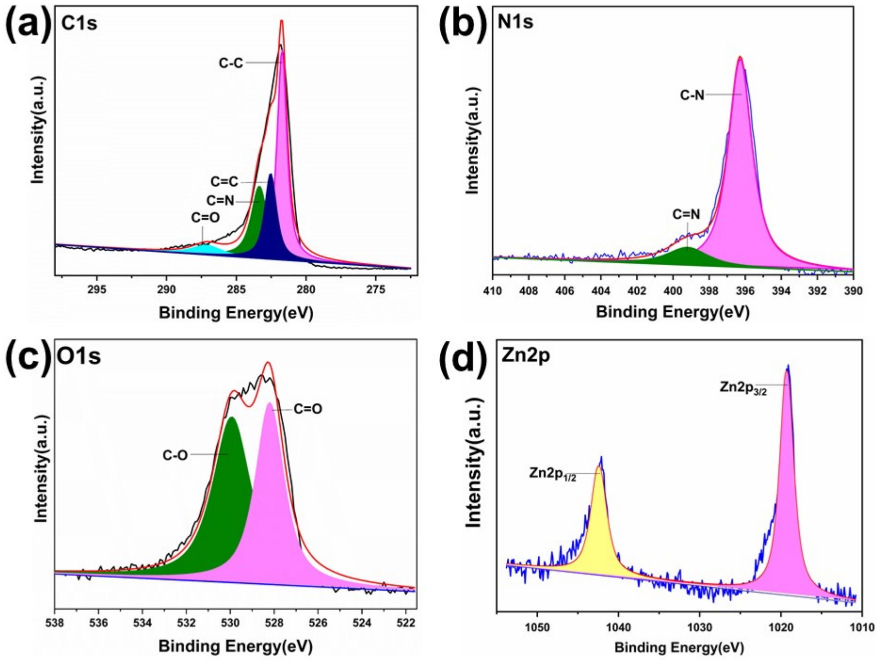

Fig. S7. High-resolution XPS spectra of Zn@COF-1 (a) Carbon atom, (b) Nitrogen atom, (c) Oxygen atom, and (d) Zn atom.

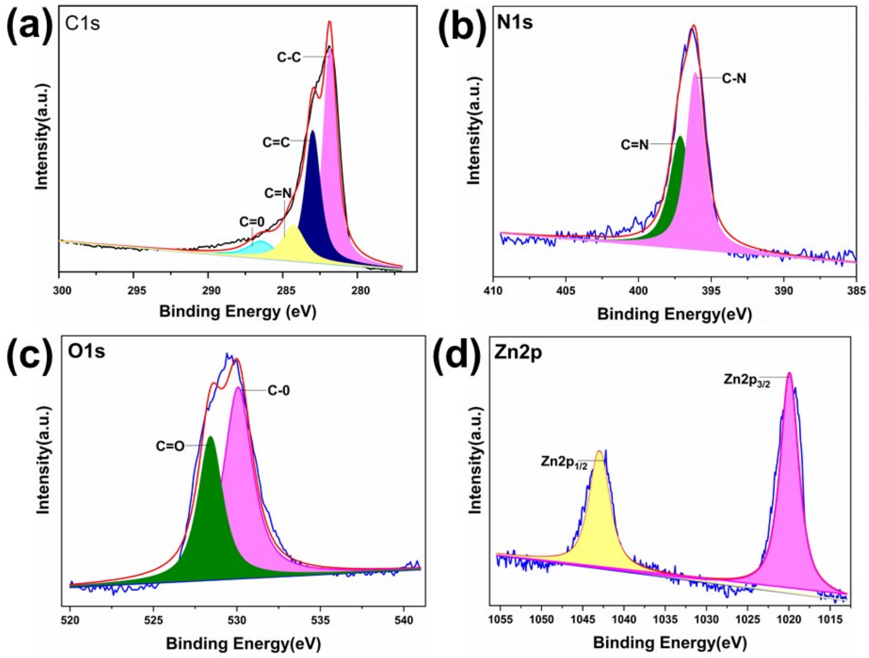

Fig. S8. High-resolution XPS spectra of Zn@COF-2 (a) Carbon atom, (b) Nitrogen atom, (c) Oxygen atom, and (d) Zn atom.

Section S-7: Brunauer–Emmett–Teller (BET) analysis

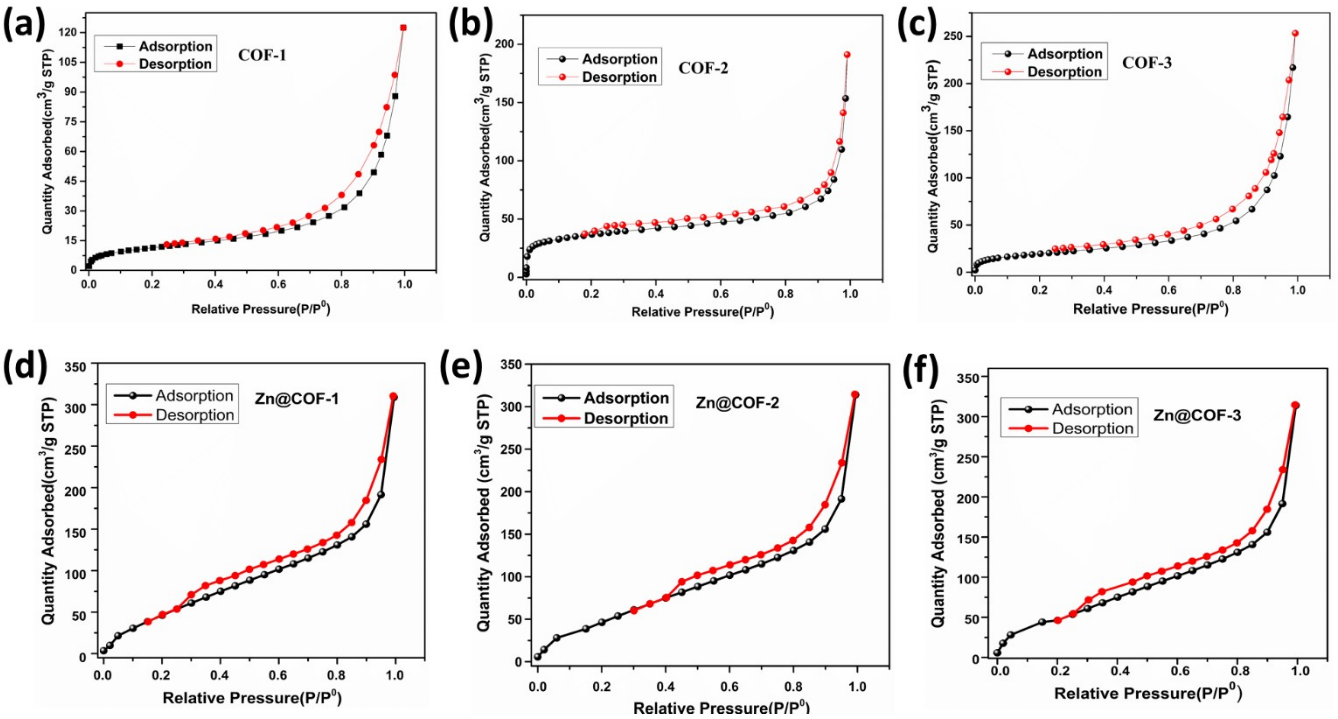

Fig. S9. N_2 - adsorption and desorption studies on (a) COF-1, (b) COF-2, (c) COF-3, (d) Zn@COF-1, (e) Zn@COF-2, and (f) Zn@COF-3.

Section S-8: Thermogravimetric Analysis (TGA)

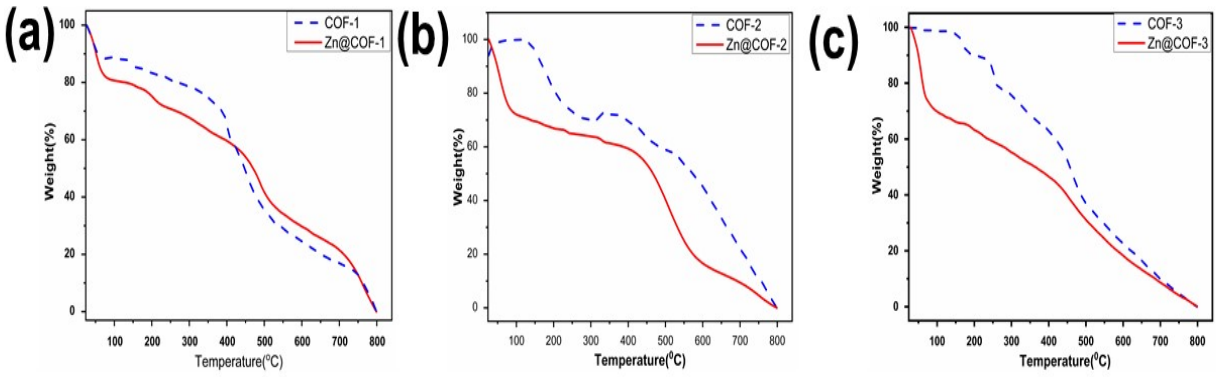

Fig. S10. TGA curves of (a) COF-1 and Zn@COF-1, (b) COF-2 and Zn@COF-2, and (c) COF-3 and Zn@COF-2.

Section S-9: Gas Sensing Responses Graphs

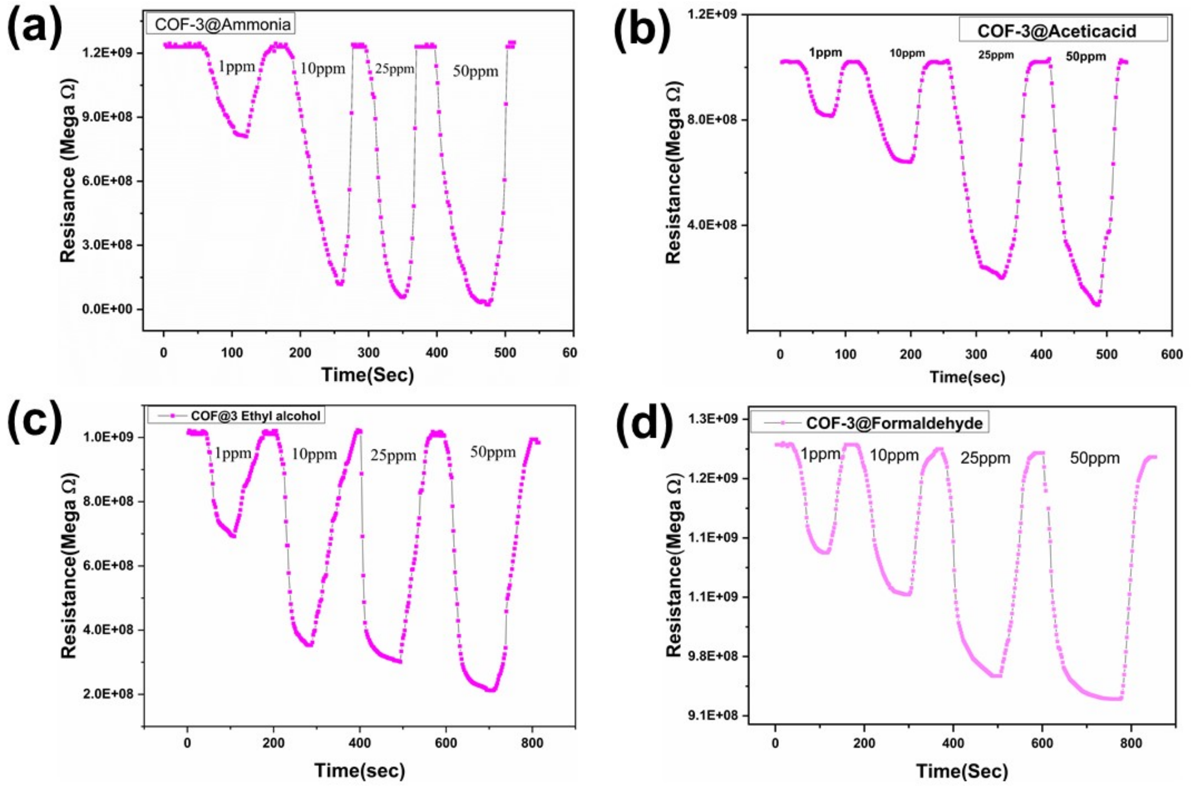

Fig. S11. Depicts the resistance curve of (a) Ammonia, (b) Acetic acid, (c) Ethyl alcohol, and (d) Formaldehyde at 1,10,25, and 50ppm concentrations of COF-3.

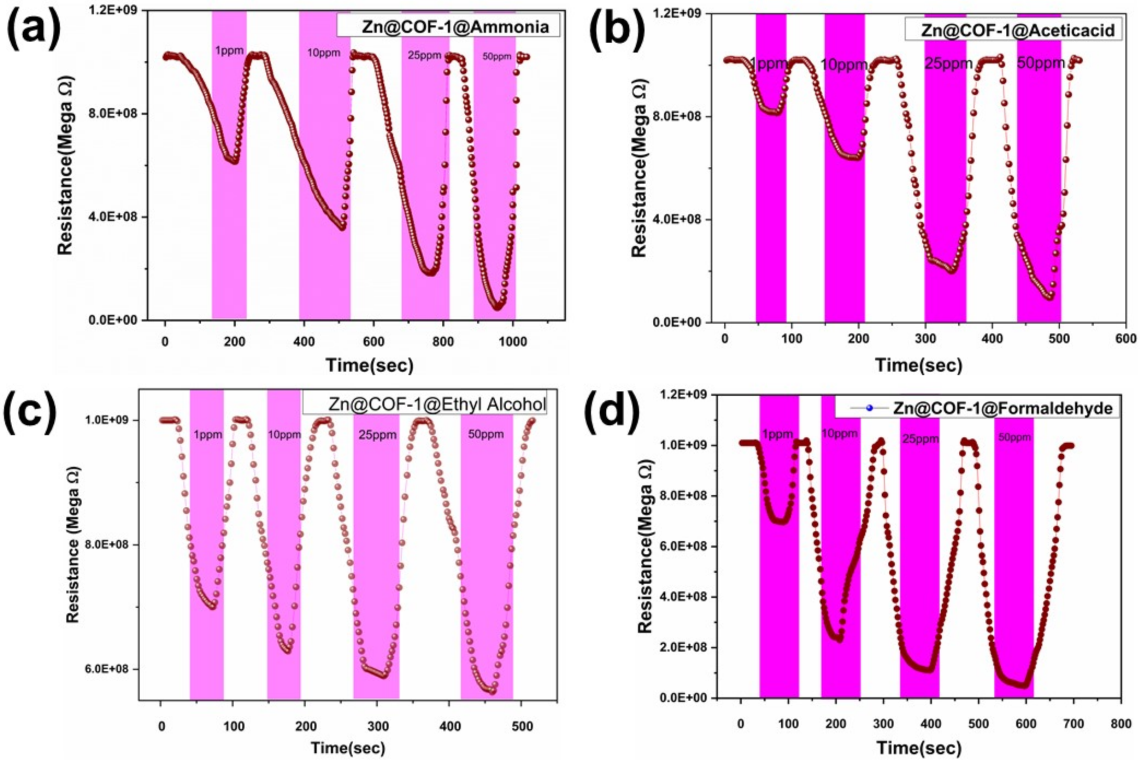

Fig. S12. Depicts the resistance curve of (a) Ammonia, (b) Ethyl alcohol, (c) Acetic acid, and (d) Formaldehyde at 1, 10, 25, and 50 ppm concentrations of Zn@COF-1.

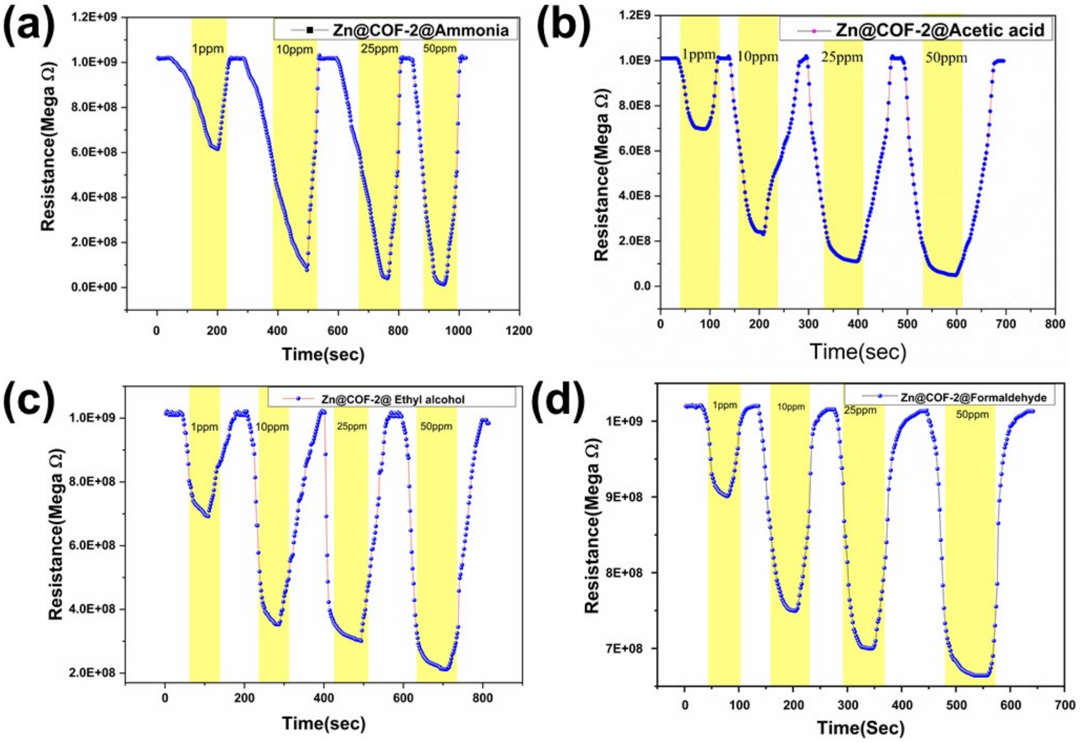

Fig. S13. Depicts the resistance curve of (a) Ammonia, (b) Ethyl alcohol, (c) Acetic acid, and (d) Formaldehyde at 1,10,25, and 50ppm concentrations of Zn@COF-2.

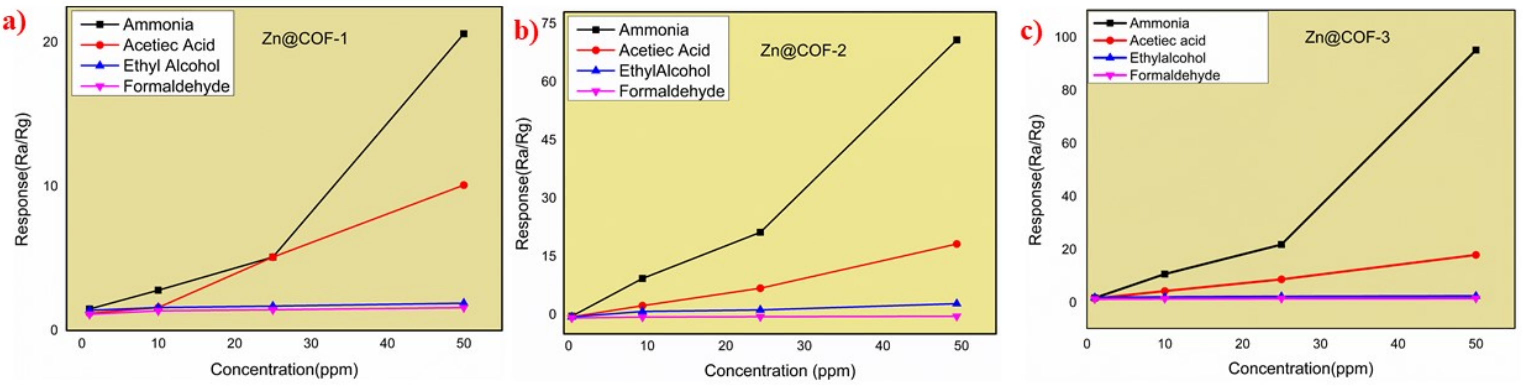

Fig. S14. The quantitative response characteristic of the as-obtained (a) Zn@COF-1, (b) Zn@COF-2, and (c) Zn@COF-3 sensors towards ammonia, acetic acid, ethanol, and formaldehyde.

Section S-10: Theoretical Studies and Results

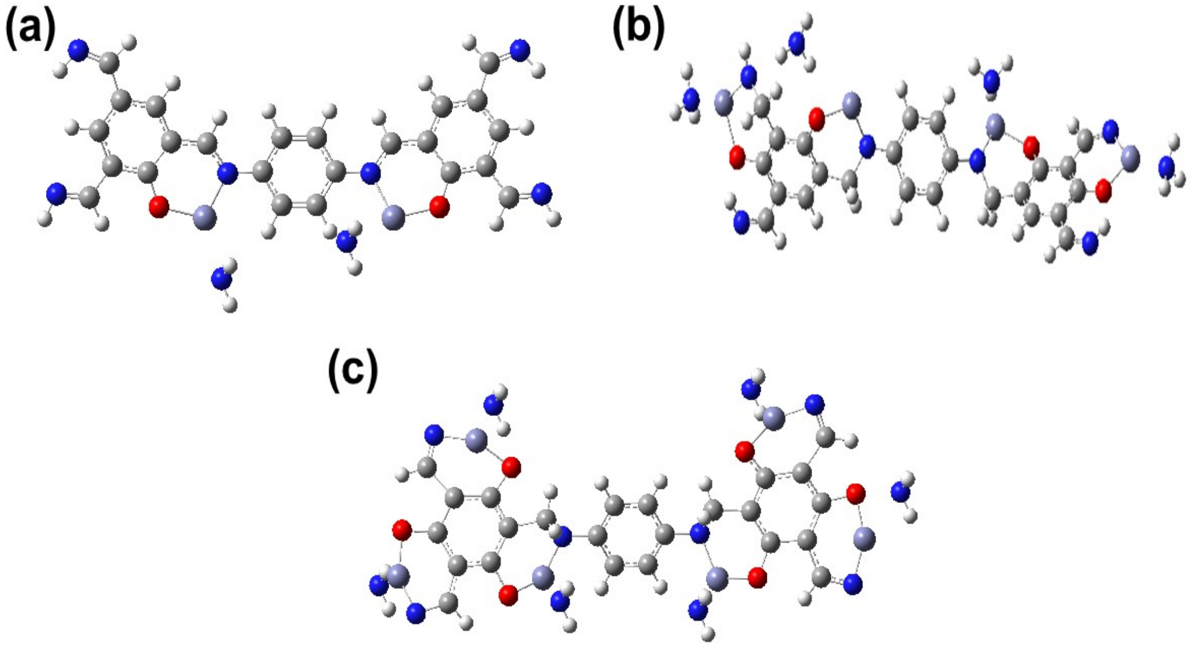

Fig. S15. Geometrical optimized structure of (a) Zn@COF-1, (b) Zn@COF-2 and (c)Zn@COF-3

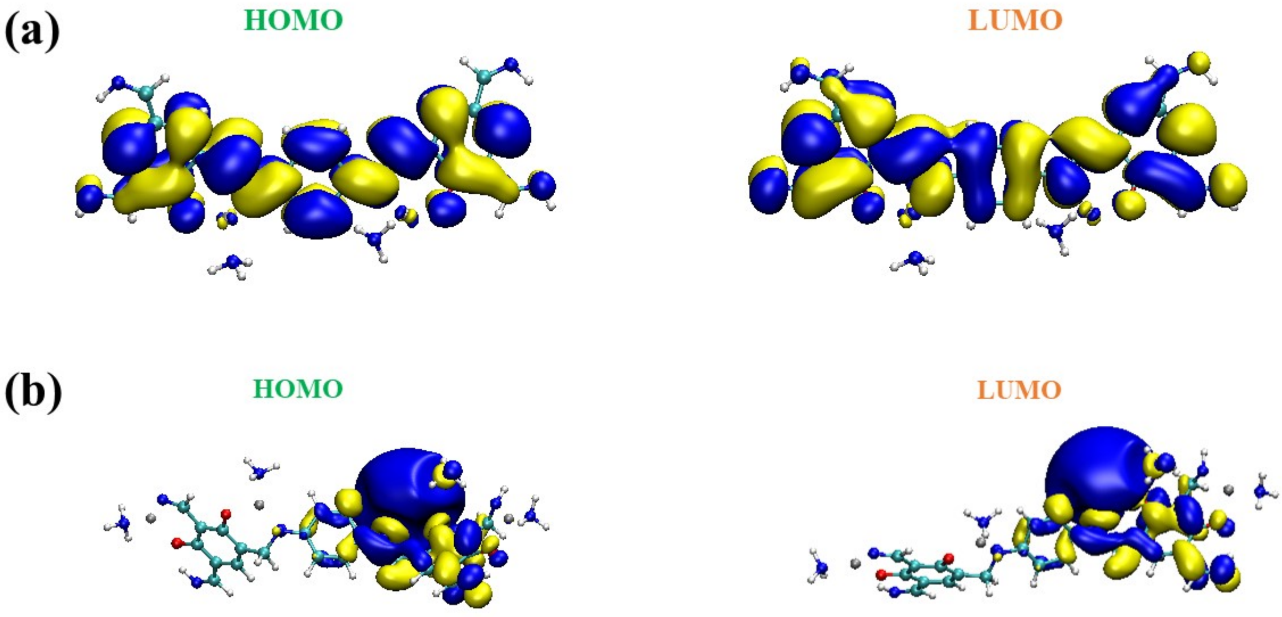

Fig. S16. HOMO and LUMO plots of (a) Zn@COF-1 and (b) Zn@COF-2.

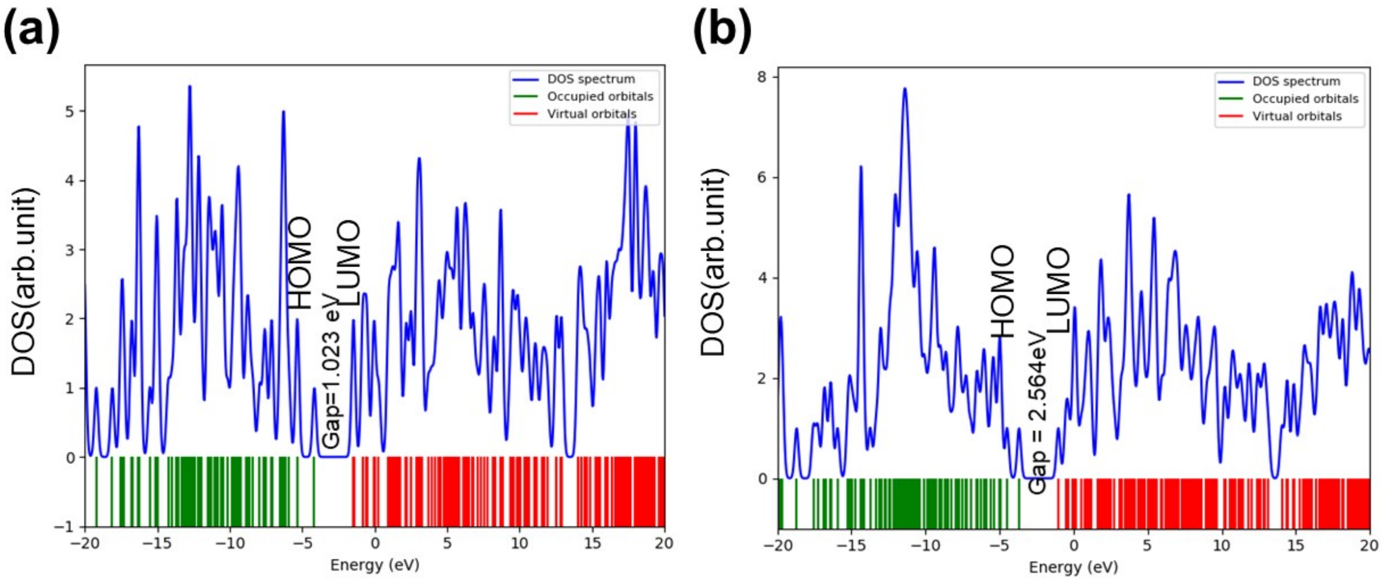

Fig. S17. Density of States (DOS) of (a) Zn@COF-1 and (b) Zn@COF-2.

Section S-11: Tables

Table S1.Calculated d-spacing and lattice parameters of parent COFs COF-1, COF-2 and COF-3.

COF Type	Plane(hkl)	Peak(2θ)	d-Spacing(A^0)	Lattice Parameters (A^0)	Space Group	Volume (A^0)
COF-1	(100)	4.8^0	18.4	a=b=23.69, c = 26.0	P1(Hexagonal)	13,303.74
COF-2	(110)	8.3^0	10.7	a=b=24.64, c = 28.0	P1(Hexagonal)	14,967.08
COF-3	(001)	26.7^0	3.34	a=b=27.05, c = 30.0	P1(Hexagonal)	19,811.08

Table S2.Calculated d-spacing and lattice parameters of Zn@COF-1, Zn@COF-2 and Zn@COF-3

COF Type	Plane(hkl)	Peak(2θ)	d-Spacing(A^0)	Lattice Parameters (A^0)	Space Group	Volume (A^0)
Zn@COF-1	(100)	4.8^0	18.4	a=b=22.00, c = 3.3	P6 (Hexagonal)	1425.00
Zn@COF-2	(110)	8.3^0	10.7	a=b=22.40, c = 3.4	P6 (Hexagonal)	1475.00
Zn@COF-3	(001)	26.7^0	3.34	a=b=22.70, c = 30.0	P6 (Hexagonal)	1520.00

Atomic Coordinates of COFs and Zn@COFs:

COF-1			
O	23.22645	10.23665	1.74167
C	22.97064	11.46887	1.72969
C	24.12047	12.49664	1.73003
C	25.45368	12.06646	1.74392
H	26.24588	12.7866	1.74446
N	23.07462	16.74358	1.6794
C	23.8079	14.00323	1.71547
C	22.34832	14.4871	1.69849
C	22.07022	15.86046	1.68298
H	21.05679	16.20297	1.67342
H	23.99679	16.49202	1.5659
N	19.19156	11.48741	1.70131
C	21.19796	13.46327	1.69847
C	21.50775	11.95394	1.71448
C	20.44994	11.03244	1.7147
C	18.15067	10.64471	1.70024
C	16.85025	11.16439	1.68994
C	18.35017	9.25572	1.70952
H	16.69858	12.22334	1.68149
H	20.64687	9.98059	1.72555
H	19.34258	8.85672	1.7166
H	19.02555	12.47293	1.69214
O	13.80262	5.75541	1.69942
N	14.87985	8.10386	1.70158

C	12.66	6.28203	1.69875
C	12.51684	7.8156	1.69745
C	13.65224	8.63592	1.69697
C	15.94359	8.91546	1.7007
C	17.24427	8.38993	1.70942
C	15.74885	10.30193	1.69069
H	17.39297	7.33057	1.71621
H	13.53596	9.69956	1.69323
H	14.7566	10.70205	1.68367
H	15.00018	7.11084	1.70587
N	7.49872	7.38652	1.69851
C	11.11795	8.45667	1.69643
C	9.8622	7.56798	1.69773
C	8.59176	8.15669	1.69765
H	8.49712	9.22227	1.69719
N	10.44102	3.22277	1.69798
C	10.00261	6.03488	1.69903
C	11.40229	5.39189	1.69939
C	11.53197	3.99715	1.70051
H	12.50417	3.55089	1.70298
H	9.53204	3.63995	1.69493
H	6.5906	7.80622	1.69839
H	7.58993	6.39067	1.6991
H	10.53509	2.22678	1.69966
H	22.70004	17.6124	1.35583
N	25.76731	10.63148	1.75799
H	26.63145	10.48385	2.23911
H	25.03619	10.13506	2.22598

H	9.48658	5.67044	2.56263
H	9.48635	5.66896	0.8362
H	24.27237	14.4306	2.57948
H	24.288	14.41716	0.85346
H	11.05913	9.08773	2.55853
H	11.05919	9.08512	0.83243
H	20.59084	13.67025	2.55489
H	20.60465	13.65478	0.82887

**COF-2**			
C	6.01611	-1.12835	0.46267
C	7.44061	-0.90115	0.60709
C	8.32049	-1.95979	0.71451
H	9.38418	-1.75014	0.80189
N	8.90776	2.43116	-1.59178
C	7.98888	0.5251	0.64978
C	7.13923	1.46218	-0.17807
C	7.59549	2.25775	-1.17035
H	6.88688	2.87532	-1.71832
H	9.66238	1.8919	-1.20025
N	2.79394	0.72108	0.1375
C	5.67517	1.44052	0.20275
C	5.11895	0.02488	0.2983
C	3.77778	-0.22882	0.24435
C	1.40714	0.49382	0.01673
C	0.53338	1.59382	0.11134
C	0.85444	-0.78258	-0.19935

H	0.93906	2.58714	0.28041
H	3.45412	-1.26056	0.3021
H	1.48886	-1.65455	-0.29998
O	-4.74565	-1.82017	-0.55379
N	-2.78088	-0.08	-0.33004
C	-5.59648	-0.84034	-0.47346
C	-5.12951	0.5377	-0.39718
C	-3.79039	0.84177	-0.3212
C	-1.40243	0.15597	-0.20836
C	-0.52566	-0.94336	-0.3048
C	-0.84642	1.43348	0.0017
H	-0.93221	-1.93563	-0.47444
H	-3.4938	1.88355	-0.2542
H	-1.47766	2.30945	0.08948
N	-9.12562	1.89061	1.96413
C	-6.15522	1.66359	-0.43946
C	-7.42368	1.28685	0.29204
C	-7.94003	2.07324	1.26205
H	-7.41225	2.98233	1.54157
N	-8.73168	-2.81912	-0.74584
C	-8.05386	-0.00627	-0.17706
C	-7.0317	-1.10175	-0.46418
C	-7.4444	-2.38695	-0.70715
H	-6.69019	-3.14479	-0.89044
H	-9.29367	2.41675	2.80506
H	-8.94711	-3.78428	-0.93523
N	7.98812	-3.2722	0.72132
H	8.69044	-3.99168	0.77426

H	-6.39363	1.89689	-1.49453
H	-3.85185	-1.47101	-0.52517
H	5.6224	-2.1232	0.47554
O	-8.97434	-0.48885	0.80515
H	-9.86968	-0.42259	0.46518
O	9.35127	0.51818	0.21534
H	9.92237	0.76153	0.94762
O	4.924	2.1974	-0.75002
H	5.04374	3.13474	-0.58077
O	10.81707	-1.66263	-0.96971
H	11.77707	-1.66263	-0.96971
H	10.49662	-0.7577	-0.96971

**COF-3**			
O	-5.66461	2.58715	0.00371
C	-6.1118	1.37826	0.00203
C	-7.53696	1.10699	0.00193
C	-8.47036	2.14426	0.00338
H	-9.52069	1.87108	0.0032
O	-9.32266	-0.48093	0.00029
N	-8.80848	-3.08129	-0.00276
C	-8.05304	-0.25871	0.00029
C	-7.10473	-1.36028	-0.00127
C	-7.53611	-2.68777	-0.00267
H	-6.77463	-3.46104	-0.00374
H	-9.51917	-2.34791	-0.00174
O	-4.8366	-2.11309	-0.00291

N	-2.85216	-0.38539	-0.0013
C	-5.66804	-1.12454	-0.00141
C	-5.18469	0.24706	0.00022
C	-3.82417	0.54099	0.00019
C	-1.45631	-0.17691	-0.00144
C	-0.62455	-1.3119	-0.00167
C	-0.86737	1.09966	-0.00144
H	-1.06781	-2.30228	-0.00179
H	-3.54545	1.58847	0.00153
H	-1.47317	1.99717	-0.00149
H	-3.21872	-1.3496	-0.00233
O	4.73433	2.02641	-0.00352
N	2.74669	0.30109	-0.00139
C	5.5631	1.03574	-0.00172
C	5.07937	-0.33465	0.0002
C	3.71845	-0.62563	0.00024
C	1.35078	0.09283	-0.0015
C	0.51901	1.22781	-0.00143
C	0.7618	-1.18373	-0.00174
H	0.96226	2.21819	-0.00135
H	3.43983	-1.67332	0.0018
H	1.36768	-2.08117	-0.00206
H	3.11326	1.2651	-0.00257
O	5.5602	-2.67391	0.00393
N	9.62883	-2.22834	0.00451
C	6.00078	-1.47491	0.00222
C	7.43574	-1.19321	0.00229
C	8.29791	-2.28981	0.00416

H	7.84426	-3.27584	0.00544
O	9.22012	0.39265	0.00068
N	8.70594	2.99414	-0.00351
C	7.94072	0.1653	0.00051
C	7.00105	1.27118	-0.00154
C	7.43414	2.59766	-0.00338
H	6.67192	3.37018	-0.00481
H	9.42069	2.26591	-0.00216
H	10.19456	-3.06344	0.00595
H	10.0523	-1.3002	0.00329
H	8.95212	3.9724	-0.00486
H	-9.05808	-4.05869	-0.00374
N	-8.17449	3.44316	0.00501
H	-8.89559	4.14853	0.00598
H	-7.18435	3.69249	0.00533

Zn@COF-1			
C	1.44644	0.07189	0.03117
C	0.69157	1.14282	0.00759
C	0.68849	-1.2832	0.01738
H	1.20165	2.0995	0.02693
H	1.21564	-2.23327	0.00172
O	-5.54307	-1.35195	-0.30848
N	-2.82969	-0.14207	-0.06159
C	-5.98561	-0.1051	-0.04494

C	-5.06565	0.99748	0.18479
C	-3.63777	0.93283	0.17563
C	-1.44642	-0.07181	-0.03138
C	-0.68852	-1.28316	-0.01757
C	-0.6915	1.14286	-0.00781
H	-1.21573	-2.23319	-0.00193
H	-3.3379	1.29816	1.13559
H	-1.20152	2.09958	-0.02709
N	-8.72564	4.28588	0.94133
C	-5.65298	2.26332	0.46437
C	-7.0388	2.47909	0.52867
C	-7.51547	3.83733	0.82847
H	-6.72072	4.5747	0.9773
N	-9.58666	-0.88979	-0.17155
C	-7.90739	1.39945	0.30544
C	-7.38987	0.12064	0.02056
C	-8.29349	-1.00596	-0.216
H	-7.79512	-1.95251	-0.43318
H	-9.4431	3.55892	0.79365
H	-10.0614	-1.78106	-0.36437
H	-8.98566	1.51262	0.34334
H	-4.98678	3.10586	0.63974
O	5.54304	-1.35201	0.30831
N	2.82973	-0.14217	0.06129
C	5.9856	-0.10513	0.04494
C	5.06565	0.9974	-0.18511
C	3.63778	0.93269	-0.17624
H	3.3381	1.29758	-1.13642

N	8.72571	4.28585	-0.94108
C	5.65301	2.26322	-0.46473
C	7.03884	2.47904	-0.52865
C	7.51553	3.83726	-0.82849
H	6.72079	4.57457	-0.97768
N	9.58664	-0.88963	0.17279
C	7.90741	1.39948	-0.305
C	7.38986	0.12068	-0.02014
C	8.29346	-1.00585	0.21685
H	7.79506	-1.95239	0.43402
H	9.44316	3.55894	-0.79312
H	10.06135	-1.78085	0.3659
H	8.98569	1.5127	-0.34257
H	4.98683	3.10571	-0.64038
Zn	-3.73092	-1.7477	-0.68513
Zn	3.73088	-1.74776	0.68497
N	-3.06725	-3.94015	-0.65525
H	-2.73393	-4.88297	-0.65525
H	-2.73391	-3.46875	0.16124
H	-2.73391	-3.46875	-1.47175
N	3.14271	-3.48113	-0.02259
H	3.47604	4.42395	0.02259
H	3.47605	-3.00973	0.79391
H	3.47605	-3.00973	-0.83908
H	3.33153	1.67172	0.53435
H	-3.3317	1.67158	-0.53534

Zn@COF-2			
C	1.41521	-0.55861	0.12645
C	0.69755	0.46577	-0.52557
C	0.69634	-1.61395	0.72308
H	1.22969	1.24125	-1.06647
H	1.2609	-2.42359	1.17564
N	-2.83745	-0.61348	0.14478
C	-5.88514	-0.39279	0.00992
C	-4.94119	0.68357	0.1628
C	-3.51481	0.50538	0.23803
C	-1.41524	-0.55873	0.12655
C	-0.69621	-1.61401	0.72315
C	-0.69773	0.46571	-0.52552
H	-1.26066	-2.42368	1.17578
H	-3.22681	0.93162	1.17626
H	-1.22998	1.24114	-1.06638
N	-8.2795	4.35788	0.20172
C	-5.43049	1.99731	0.23936
C	-6.77951	2.34616	0.15794
C	-7.12589	3.76924	0.24909
H	-6.25352	4.42002	0.37533
N	-9.46999	-1.22646	-0.41384
C	-7.74655	1.30262	-0.01387
C	-7.30659	-0.06337	-0.1009
C	-8.15496	-1.19337	-0.2896
H	-7.61404	-2.13721	-0.3341

H	-9.03643	3.67046	0.08179
H	-4.70806	2.80214	0.36478
N	2.83743	-0.61325	0.14467
C	5.88503	-0.39259	0.00949
C	4.94114	0.68381	0.16255
C	3.51474	0.50563	0.23771
H	3.22666	0.93209	1.17581
N	8.27964	4.35795	0.20213
C	5.4305	1.9975	0.23926
C	6.77956	2.34629	0.15795
C	7.12601	3.76935	0.2493
H	6.25366	4.42013	0.37566
N	9.46984	-1.2265	-0.41396
C	7.74654	1.30272	-0.01393
C	7.30651	-0.06325	-0.10114
C	8.1548	-1.19329	-0.28989
H	7.78046	-1.67849	-1.16703
H	9.03655	3.67051	0.08218
H	9.87038	-2.14932	-0.54813
O	-9.03388	1.65929	-0.08083
O	9.03391	1.65934	-0.08078
O	-5.50119	-1.63744	-0.01586
Zn	10.43747	0.40638	-0.30529
Zn	-3.74035	-2.31578	0.12321
Zn	-10.4376	0.40647	-0.30536
O	5.50099	-1.63719	-0.01656
Zn	3.75515	-2.36817	0.1237
H	4.70811	2.80236	0.36475

H	3.1109	1.14324	-0.52075
H	-3.11091	1.14318	-0.52025
H	7.93638	-1.84494	0.5302
N	12.64557	1.45435	-1.61983
H	12.97889	0.51154	-1.61983
H	12.97891	1.92575	-0.80334
H	12.97891	1.92575	-2.43633
N	2.55817	-4.65652	-1.24519
H	2.8915	-5.59934	-1.24519
H	2.89151	-4.18512	-0.4287
H	2.89151	-4.18512	-2.06169
N	-2.80771	-4.39916	-0.39754
H	-2.47439	-5.34197	-0.39754
H	-2.47437	-3.92776	0.41896
H	-2.47437	-3.92776	-1.21403
N	-12.6022	-0.29852	1.82027
H	-12.2689	-1.24134	1.82027
H	-12.2689	0.17288	2.63677
H	-12.2689	0.17288	1.00378

Zn@COF-3			
C	1.39642	-1.54256	0.45724
C	0.7067	-0.66928	1.32618
C	0.58597	-2.43235	-0.29748
H	1.26019	0.01578	1.96002
H	1.07553	-3.12212	-0.98379

N	-2.9024	-1.6081	0.70807
C	-5.9668	-1.07801	0.46707
C	-4.96643	-0.13231	0.74833
C	-3.64411	-0.52184	1.39747
C	-1.50402	-1.55017	0.66259
C	-0.79877	-2.434	-0.20244
C	-0.69752	-0.67412	1.42619
H	-1.37217	-3.11634	-0.82608
H	-3.8219	-0.78954	2.45943
H	-1.15713	0.00401	2.13761
C	-5.21441	1.24918	0.48848
C	-6.45798	1.70366	-0.07124
C	-6.81273	3.09238	-0.40376
N	-9.45281	-1.46291	-0.88762
C	-7.48427	0.72217	-0.35307
C	-7.23728	-0.66819	-0.0848
C	-8.19172	-1.69244	-0.34779
H	-7.83965	-2.69103	-0.08533
N	2.78879	-1.58975	0.3033
C	5.96377	-1.34815	0.20008
C	5.01881	-0.34609	0.44526
C	3.62917	-0.60509	1.03022
H	3.74241	-0.89848	2.09461
C	5.3937	1.02763	0.2403
C	6.73236	1.39502	-0.14026

C	7.19879	2.79017	-0.31418
N	9.46113	-2.16267	-0.73958
C	7.6942	0.36646	-0.34083
C	7.32573	-1.0218	-0.19558
C	8.17156	-2.1366	-0.41269
H	7.67049	-3.09261	-0.28012
O	-8.76495	1.04297	-0.89999
O	8.94774	0.72761	-0.68448
O	4.45309	1.95532	0.43806
O	-5.77446	-2.39891	0.70715
Zn	10.34631	-0.49982	-0.92751
Zn	-3.99874	-3.10216	0.45254
Zn	-10.3796	0.17199	-1.47281
O	5.67565	-2.67718	0.32788
O	-4.21028	2.10885	0.81382
Zn	3.86538	-3.05265	-0.05044
H	8.27304	2.80964	-0.54064
H	-7.81962	3.12032	-0.84068
N	-6.20314	4.25234	-0.29368
N	6.57971	3.93229	-0.24311
Zn	-4.51452	3.93822	0.48013
Zn	4.72971	3.85663	0.12892
H	3.12705	0.36536	1.03534
H	-3.03898	0.38669	1.4116
N	11.40743	-0.01991	0.89508

H	11.74075	-0.96272	0.89508
H	11.74077	0.45149	1.71158
H	11.74077	0.45149	0.07858
N	-2.75191	5.48007	0.01541
H	-2.41859	4.53726	0.01541
H	-2.41857	5.95147	0.83191
H	-2.41857	5.95147	-0.80108
N	3.09144	6.10337	0.01541
H	3.42477	5.16055	0.01541
H	3.42478	6.57477	0.83191
H	3.42478	6.57477	-0.80108
N	3.20831	-4.999	0.01541
H	3.54163	-5.94182	0.01541
H	3.54165	-4.5276	0.83191
H	3.54165	-4.5276	-0.80108
N	-3.29729	-5.15483	0.01541
H	-2.96397	-6.09764	0.01541
H	-2.96395	-4.68343	0.83191
H	-2.96395	-4.68343	-0.80108
N	-13.0362	0.1821	0.01541
H	-12.7029	-0.76071	0.01541
H	-12.7029	0.6535	0.83191
H	-12.7029	0.6535	-0.80108

Target Gas	Concentration (PPM)	Sensing Material: COF-1			Sensing Material: COF-2			Sensing Material: COF-3		
		Response (Ra/Rg)	Response time(Sec)	Recovery time(Sec)	Response (Ra/Rg)	Response time(Sec)	Recovery time(Sec)	Response (Ra/Rg)	Response time(Sec)	Recovery time(Sec)
Ammonia	1	1.02	118	42	1.2	110	40	1.5	38	22
Ammonia	10	1.63	175	64	9.8	177	61	10.5	43	35.6
Ammonia	25	4.45	163	58	14.14	136.1	49.4	21.6	39	33.2
Ammonia	50	11.65	116	48	23.88	79	46	55.8	34	29
Acetic acid	1	Not detected	-	-	1.35	29.82	23.49	1.34	35.67	23
Acetic acid	10	Not detected	-	-	4.2	36.65	68.65	4.08	54	29
Acetic acid	25	Not detected	-	-	8.1	42.7	56.16	7.64	29.8	13
Acetic acid	50	Not detected	-	-	17.24	40.99	50.62	15.80	27.23	15
Ethyl alcohol	1	1.12	42	33.8	Not detected	-	-	1.2	49.37	19
Ethyl alcohol	10	1.50	59	37.66	Not detected	-	-	1.26	59.93	29.3
Ethyl alcohol	25	1.63	58	38.04	Not detected	-	-	1.29	51.72	28.1
Ethyl alcohol	50	1.89	48	36.49	Not detected	-	-	1.47	48.12	30.24
Formaldehyde	1	Not detected	-	-	Not detected	-	-	1.13	28.3	26
Formaldehyde	10	Not detected	-	-	Not detected	-	-	1.16	51.8	33.6
Formaldehyde	25	Not detected	-	-	Not detected	-	-	1.19	27.49	65.1
Formaldehyde	50	Not detected	-	-	Not detected	-	-	1.26	44.1	38.9

Table S3. Response, response times, and recovery times for COF-1, 2, and 3 compounds for different concentrations of Ammonia, Acetiec acid, Formaldehyde, and Ethanol.

Target Gas	Concentration (ppm)	Sensing Material: Zn@COF-1			Sensing Material: Zn@COF-2			Sensing Material: Zn@COF-3		
		Response (R_a/R_g)	Response time (Sec)	Recovery Time(sec)	Response (R_a/R_g)	Response Time(sec)	Recovery Time(sec)	Response (R_a/R_g)	Response time (Sec)	Recovery Time(sec)
Ammonia	1	1.65	105	31	1.65	100	29	2.54	26	18
Ammonia	10	2.83	163	24	11.33	167	34.47	11.58	28	12
Ammonia	25	5.54	121	19	23.18	125	31.24	36.13	25	8
Ammonia	50	20.64	53	28.5	72.85	66	40.64	94.90	17	6
Acetic Acid	1	1.248	37	27.8	1.44	25.76	17.29	1.54	31.7	17
Acetic Acid	10	1.588	39	36.3	4.34	37.65	65.65	4.28	46	22
Acetic Acid	25	5.100	65	75.4	8.84	39.7	55.16	8.54	26.8	10
Acetic Acid	50	10.09	60	43.1	20.24	39.99	62.62	17.80	23.2	8
Ethyl Alcohol	1	1.42	30	23.8	1.46	30.25	23.64	1.42	42.37	12
Ethyl Alcohol	10	1.58	39	26.46	2.87	43.4	32.3	1.47	56.93	24.3
Ethyl Alcohol	25	1.69	41	29.04	3.21	44.97	34	1.57	48.32	24.1
Ethyl Alcohol	50	1.76	57	40.49	4.78	58.94	42.22	1.9	50.97	31.24
Formaldehyde	1	1.13	17	13.2	1.13	17.4	17.3	1.11	25.3	21
Formaldehyde	10	1.37	37	13.58	1.36	31.5	25.1	1.23	41.8	23.6
Formaldehyde	25	1.45	41	26.46	1.45	22.7	30.43	1.28	20.9	53.1
Formaldehyde	50	1.59	49	28.94	1.56	30.5	22.89	1.36	39.1	30.9

Table S4. Response, response time, and recovery time for Zn@COF-1, 2, and 3 compounds for different concentrations of Ammonia, Acetic acid, Formaldehyde, and Ethanol.

	E_{HOMO}	E_{LUMO}	Egap
COF-1	-2.781	-1.76	1.021
Zn@COF-1	-4.161	-1.485	2.676
COF-2	-3.301	-1.917	1.384
Zn@COF-2	-3.219	-0.882	2.337
COF-3	-2.572	-1.363	1.209
Zn@COF-3	-3.681	-1.031	2.65

Table S5. HOMO energies(E_{HOMO}) LUMO energies(E_{LUMO}) and energy gap calculated for COF-1,2and 3and Zn@COF-1,2 and 3.

	Energy eV	$E_{ads\ (ev)}$
NH_3	-1538.3	
COF-1	-135028.9	
Zn@COF-1	-138139.6	-1572.5
COF-2	-235915.6	
Zn@COF-2	-242107.9	-4654.1
COF-3	-336801.9	
Zn@COF-3	-346005.7	-7665.5

Table S6. The total energy and absorption energy are computed from COF-1, 2, and 3 and Zn@COF1, 2, and 3.
